# Effects of Citrulline Malate Supplementation on Exercise Performance: A Systematic Review and Three-Level Meta-Analysis

**DOI:** 10.3390/nu18121881

**Published:** 2026-06-11

**Authors:** Xuexiang Wang, Xiaohan Fan, Jindong Chang, Hansen Li, Xing Zhang, Yilin Zhang, Tianyu Song, Ping Liu, Qing Huang, Mohamed Nashrudin Bin Naharudin, Hengzhi Deng

**Affiliations:** 1 College of Aviation Electronics and Electrical Engineering, Civil Aviation Flight University of China, Deyang 618307, China; 2 Faculty of Sports and Exercise Science, University Malaya, Kuala Lumpur 50603, Malaysia; 3 School of Physical Education, Southwest University, Chongqing 400715, China; 4 School of Physical Education, Sichuan Agricultural University, Ya’an 625014, China; 5 Department of Physical Education and Sport, Faculty of Sport Sciences, University of Granada, 18071 Granada, Spain; 6 School of Sports Training, Tianjin University of Sport, Tianjin 301617, China

**Keywords:** citrulline malate, ergogenic aid, exercise performance, meta-analysis

## Abstract

**Background:** Citrulline malate (CM) is commonly used as an ergogenic supplement, but its effects on exercise performance and perceived exertion remain uncertain. This systematic review and meta-analysis evaluated the effects of CM supplementation, with attention paid to differences between acute and chronic protocols. **Methods:** Six databases were searched from inception to August 2025. Randomized controlled trials examining CM supplementation on exercise performance and/or perceived exertion were included. Hedges’ g was synthesized using three-level random-effects models to account for dependent effect sizes. Subgroup and moderator analyses explored supplementation protocol, exercise modality, sex, training status, dosage, and ingestion timing. Risk of bias, small-study effects, sensitivity analyses, and GRADE certainty were assessed. **Results:** Thirty randomized controlled trials contributed 138 effect sizes from 644 participants. CM supplementation was associated with a small improvement in overall exercise performance (g = 0.16, *p* = 0.01); however, prediction intervals were wide and statistical power was limited. The pooled effect on perceived exertion was not statistically significant. Current evidence appeared more stable for acute than chronic supplementation, although the protocol subgroup difference remained uncertain. Among acute studies, exploratory subgroup analyses suggested possible benefits for aerobic endurance and short anaerobic tasks, but these findings were not robust across sensitivity analyses. No significant between-subgroup differences were found for sex, training status, dosage, or ingestion timing. GRADE certainty ranged from low to very low. **Conclusions:** CM supplementation may be associated with small, context-dependent improvements in exercise performance, but current evidence remains limited and uncertain. Reliable dosing, timing, and target populations have not been established. Larger trials with verified supplement composition and standardized protocols are needed.

## 1. Introduction

Citrulline malate (CM) is a popular dietary supplement marketed as an ergogenic aid for exercise performance [1]. CM is the salt of L-citrulline, a non-essential amino acid, and malate, an intermediate of the tricarboxylic acid cycle [2]. The rationale for CM centers on complementary actions. As a precursor to L-arginine, L-citrulline can increase nitric oxide availability through the L-arginine–nitric oxide (NO) pathway, which may enhance vasodilation, muscle oxygen delivery, and substrate transport during exercise [3,4]. L-citrulline also participates in ammonia buffering via stimulation of the urea cycle, which could mitigate fatigue during high-intensity or prolonged efforts [5,6]. Malate may support oxidative metabolism by feeding the tricarboxylic acid cycle (TCA) cycle, thereby sustaining ATP production during exercise [2]. These mechanisms suggest the potential for additive or synergistic effects on performance.

Empirical findings, however, have been mixed across controlled trials involving resistance, endurance, and mixed exercise tasks, with substantial variation in CM dosage, supplementation timing, intervention duration, and formulation. Some studies have reported improvements in muscular endurance or time-trial performance [7,8,9], whereas others have observed no ergogenic benefits [10,11,12]. Interpretation is further complicated by previous evidence syntheses that pooled studies of pure L-citrulline with CM, thereby limiting conclusions specific to CM supplementation [1,13,14].

To date, only two quantitative syntheses have specifically evaluated the effects of CM supplementation on exercise performance. Vårvik et al. (2021) examined muscular endurance during resistance exercise and reported a small ergogenic effect on total repetitions performed (Hedges’ g = 0.196) based on eight acute trials without evaluating outcomes such as maximal strength, anaerobic capacity, or aerobic performance [15]. In contrast, Aguiar & Casonatto (2022) [16] focused exclusively on maximal muscular strength in resistance-trained adults incorporating four trials without distinguishing between acute and chronic supplementation protocols, and found no performance benefits. These reviews therefore reach divergent conclusions and each addresses only a narrow aspect of the available evidence [16]. Beyond these two meta-analyses, the literature on CM consists predominantly of narrative reviews and qualitative summaries, which often emphasize mechanistic considerations but provide limited synthesis of trial-based outcomes [17,18].

Therefore, the current evidence gap is not only whether CM has an overall effect on exercise performance, but also whether potential effects are more apparent under specific conditions. In particular, it remains unclear whether acute and chronic supplementation protocols should be interpreted together, whether effects vary across broader exercise domains, and whether multiple dependent outcomes extracted from the same studies influence the precision of pooled estimates. A CM-specific synthesis that addresses these issues may provide a more transparent assessment of the available evidence and support more cautious evidence-based decisions for athletes, coaches, and practitioners. Accordingly, we conducted this systematic review and three-level meta-analysis to provide an up-to-date synthesis of CM supplementation and exercise performance. The specific aims were to quantify the overall effect of CM, compare acute and chronic supplementation protocols, account for dependent effect sizes using a three-level model, and classify performance outcomes across broader exercise domains, including aerobic endurance, anaerobic performance, strength/power, and muscular endurance. Prespecified moderator analyses examined sex, training status, exercise domain, CM formulation, supplementation timing, and dosage, with dosage additionally evaluated as a continuous predictor in meta-regression. This analysis also prespecified perceived exertion as a secondary outcome to contextualize the performance findings.

## 2. Methods

The systematic review adhered to the 2020 Preferred Reporting Items for Systematic Reviews and Meta-Analyses (PRISMA) statement [19]. The completed PRISMA 2020 checklist is available in Electronic Supplementary Material S1. Additionally, this review protocol was preregistered with the Open Science Framework (OSF; registration ID: osf.io/tez3a) on 6 August 2025. The full extraction and effect-size calculation dataset used for the analyses, including raw numerical data, assumed correlations, Hedges’ g, standard errors, variances, and effect-direction coding, as well as the detailed RoB 2 assessment records, is available in the same OSF project.

### 2.1. Eligibility Criteria

For this systematic review and meta-analysis, studies were included if they met the following inclusion criteria: (1) randomized controlled trials published as full-text articles in peer-reviewed journals; (2) investigated the effects of CM supplementation, either as the sole active ingredient differing between intervention and control conditions or as a separately extractable CM-only arm; (3) included at least one measure of exercise performance derived from a structured exercise test; (4) involved original research on human subjects; and (5) included healthy adult participants without known clinical conditions (≥18 years of age). Studies were excluded if they met any of the following criteria: (1) the intervention involved L-citrulline without malate and did not include a separately extractable CM arm; (2) CM was co-administered with other active substances exclusively in the experimental group, such that the independent effect of CM could not be isolated; (3) the experimental group underwent additional exercise training not equally applied to the control group; (4) animal or in vitro studies; (5) no exercise performance outcomes were reported; (6) not original research (e.g., reviews, trial protocols, conference abstracts); (7) not published in English; or (8) lacked sufficient methodological detail to assess eligibility.

Non-active vehicles or matched co-interventions, such as carbohydrate, flavoring agents, juice, or similar placebo components, were permitted when provided equally to both CM and placebo/control conditions, because the between-condition contrast isolated the effect of CM. In multi-arm studies, only eligible CM-only contrasts were extracted; arms involving co-supplementation with additional active ergogenic substances were excluded unless the same co-intervention was matched in the control condition.

Because the primary objective was to evaluate the effects of CM on objective exercise performance, studies were eligible for quantitative synthesis only if they reported at least one exercise performance outcome. Subjective perceptual outcomes, including rating of perceived exertion (RPE) or visual analog scale (VAS) scores, were extracted as secondary outcomes when available, but studies only reporting perceptual outcomes without exercise performance measures were excluded.

### 2.2. Data Sources and Search

The final systematic search was conducted on 13 August 2025 across PubMed, Web of Science, Cochrane Library, Embase, SciELO, and SPORTDiscus. The same core Boolean search string was entered into each database using the default search field settings of the respective platform: (“citrulline malate” OR “citrulline-malate” OR “L-citrulline malate” OR “L-citrulline DL-malate” OR (citrulline AND malate)) AND (“exercise performance” OR endurance OR “aerobic capacity” OR “anaerobic capacity” OR strength OR “resistance training” OR VO2max OR fatigue OR “time to exhaustion”).

No publication year restrictions or additional database filters were applied. The complete search records for each database are provided in Supplementary Material S2.

### 2.3. Data Extraction

All retrieved titles and abstracts were downloaded into Microsoft Excel spreadsheets and EndNote (21) and manually cross-referenced to identify duplicates. Article screening was performed independently by two researchers (D.H.Z and S.T.Y), and discrepancies were resolved by consensus.

Data extracted included:

(1) Study characteristics: authors, publication year, study design (parallel or crossover);
(2) Participant characteristics: sample size, sex, age, training status, and health status;
(3) Intervention characteristics: type of CM (solid or liquid), dosage, supplementation duration and timing relative to exercise;
(4) Exercise testing protocols;
(5) Primary outcomes related to exercise performance: VO_2_max or VO_2_peak, time to exhaustion, anaerobic power (e.g., peak or mean power), muscular strength (e.g., 1RM, bench press), muscular endurance (e.g., push-up test, reps to failure), and sport-specific performance tests (e.g., sprint time, jump height, agility tests);
(6) Secondary outcomes: self-reported fatigue scores. To improve between-study comparability, perceptual outcomes assessed at multiple time points were extracted from a standardized workload or the final completed stage of exercise whenever possible.

When data were not reported numerically, authors were contacted or WebPlotDigitizer (v4.8; (https://apps.automeris.io/wpd/, accessed on 15 August 2025)) was used for extraction [20].

### 2.4. Quality and Risk of Bias Assessment

The methodological quality of the included studies was evaluated using a modified version of the Physiotherapy Evidence Database (PEDro) scale. In accordance with prior recommendations [21], the original PEDro checklist was adapted to better suit the specific aims of this review. An additional item (Item 12) was incorporated: “Did the study assess the effectiveness of placebo blinding?” The first item is for descriptive purposes only and does not contribute to the total score. As a result, the maximum attainable score was 11. Studies scoring 10–11 were classified as having excellent methodological quality, 7–9 as good, 5–6 as fair, and those scoring below 5 were considered to be of poor quality.

Quality assessments were independently performed by one reviewer (D.H.Z.) and subsequently cross-verified by a second reviewer (S.T.Y.). Any discrepancies in scoring were resolved through discussion until consensus was reached.

In parallel, risk of bias was assessed using the Cochrane Collaboration Risk of Bias Tool 2 (Rob2) [22]. For parallel-group trials, the standard RoB 2 domains were assessed: bias arising from the randomization process, bias due to deviations from intended interventions, bias due to missing outcome data, bias in measurement of the outcome, and bias in selection of the reported result. For crossover trials, the RoB 2 crossover version was used, including the additional domain for bias arising from period and carryover effects. Each domain and overall risk of bias were rated as low risk, some concerns, or high risk. Assessments were performed independently by the same two reviewers, with disagreements resolved in the same manner.

### 2.5. Statistical Analysis

#### 2.5.1. Data Extraction, Synthesis and Effect Measures

All effect size calculations followed the recommendations of the Cochrane Handbook for Systematic Reviews of Interventions (2nd ed., 2019). Because most included trials had relatively small sample sizes, standardized mean differences were estimated using Hedges’ g with correction for small-sample bias [23].

Because both within-subject crossover and parallel-group designs were included, effect sizes were calculated according to study design. For crossover trials, the paired-sample structure was accounted for by incorporating the within-participant correlation (r) between CM and placebo/control conditions when estimating the sampling variance for paired contrasts [24]. When change-score data were used, the pre–post correlation within each condition was required to derive the standard deviation or variance of change scores when these values were not directly reported. For parallel trials, Hedges’ g was computed from between-group differences using post-intervention values or change scores as reported [25].

For multi-arm studies with more than one eligible CM intervention group sharing the same control condition, separate effect sizes were calculated for each eligible comparison. Shared control groups were not treated as independent studies, and their participant numbers were counted only once in descriptive summaries. Sample sizes from shared controls were not split; instead, dependence among effect sizes from the same study was handled by nesting effect sizes within studies in the three-level meta-analytic model.

Because most included studies did not report the required correlation coefficients, a moderate correlation of r = 0.50 was assumed for the primary analyses, in accordance with commonly used approaches for handling unavailable correlation parameters in paired or repeated-measures meta-analytic data [26]. The same set of assumed correlation values was applied to missing within-participant treatment correlations and missing pre–post correlations to maintain a consistent and comparable calculation framework across studies. Sensitivity analyses were conducted using lower and higher correlation values (r = 0.20 and r = 0.80) to evaluate the robustness of the findings.

For subjective fatigue outcomes, data extraction and synthesis followed the same procedures as those applied to exercise performance outcomes.

Effect sizes were interpreted using conventional thresholds: trivial (<0.2), small (0.2–0.5), moderate (0.5–0.8), and large (>0.8) [27]. Detailed computational formulas and derivations are provided in Supplementary Material S3.

#### 2.5.2. Meta-Analysis and Heterogeneity

To account for dependency arising from multiple outcomes or contrasts reported within individual studies, three-level meta-analytic models were implemented using the *rma.mv* function in the *metafor* package in R. The model used Hedges’ g as the observed effect size and its sampling variance as the known level-1 variance. Effect sizes were nested within studies using a random-effects structure equivalent to *random =* ~ 1|*study*/*effect_size*, allowing estimation of both within-study heterogeneity among effect sizes (level 2) and between-study heterogeneity (level 3). Thus, variance was partitioned into sampling variance, within-study variance, and between-study variance [28].

Model parameters were estimated using restricted maximum likelihood (REML), and robustness was examined by comparing results with maximum likelihood (ML) estimation [29,30,31]. Tests of individual coefficients and their corresponding confidence intervals (CI) were calculated using the t-distribution [32]. Prediction intervals (PI) were also calculated to estimate the range of true effects expected in comparable future studies [33,34]. Cluster-robust variance estimation was considered but not additionally applied, because dependency among effect sizes was modeled directly within the prespecified three-level framework and robustness was evaluated through REML/ML comparisons and sensitivity analyses.

Between-study heterogeneity was quantified using the *I*^2^ statistic, interpreted as low (0–25%), moderate (25–49%), substantial (50–74%), or considerable (≥75%) [35]. In addition, post hoc statistical power was estimated using the *metameta* package [36]. Because observed power is closely related to effect estimates and their precision, these estimates were not used as a basis for inference and were interpreted only alongside confidence intervals, prediction intervals, and certainty-of-evidence assessments.

#### 2.5.3. Moderators and Subgroup Analysis

To explore between-study heterogeneity and identify conditions under which CM supplementation is most likely to influence exercise performance, moderator and regression analyses were conducted for the primary outcome [37]. In line with methodological guidance, a minimum of ten studies was considered advisable for meta-regression and at least five studies per subgroup for valid subgroup analyses [38,39].

To examine whether supplementation protocol influenced overall effect estimates, a primary subgroup analysis was conducted comparing acute single-dose versus multi-day supplementation regimens. All subsequent analyses were restricted to acute supplementation studies. This decision reflected the pharmacokinetic differences between single-dose and sustained supplementation protocols, as well as the predominance of acute studies in the dataset, thereby improving interpretability and consistency.

The evaluated moderators for acute studies included: (1) sex group (male, female, mixed); (2) training status; (3) CM form (solid or liquid); (4) exercise category; (5) CM dosage (primary analysis as categorical subgroups, with the originally reported total CM dose [g] also tested as a continuous exploratory variable in meta-regression); and (6) supplementation timing (analyzed categorically only, due to the narrow distribution and small number of studies in the timing categories).

Based on previous participant categorization frameworks, participants were classified as untrained and trained (recreationally active, trained/developmental, well-trained/national level, elite/international level, and world-class) [40]. To better explore the potential ergogenic effects of CM and to reduce the instability of the computational model due to the small sample size, performance outcomes were grouped into four physiological domains based on the primary potential energy system or neuromuscular mechanism [41,42]: (1) Strength/Power: Efforts lasting ≤10 s, characterized by maximal intensity and primarily relying on the ATP–phosphocreatine system (e.g., vertical jump height, 1–3 RM lifts, maximal short sprints); (2) Anaerobic Performance: Continuous efforts lasting >10 s and ≤60 s, predominantly dependent on anaerobic glycolysis (e.g., 30-s Wingate test, repeated sprint ability); (3) Muscular Endurance: Sustained submaximal contractions performed to volitional exhaustion or failure, typically involving repeated or continuous efforts targeting a specific muscle group, and generally lasting from ~30 s to several minutes depending on protocol (e.g., bench press to failure at submaximal load, repeated knee extensions to exhaustion); (4) Aerobic Endurance: Continuous efforts exceeding ~2–3 min (typically >10 min) where oxidative metabolism provides the predominant energy supply, even when significant anaerobic contributions occur in early phases (e.g., 6 × 300-m all-out swimming test, VO_2_max ramp protocols, time to exhaustion treadmill tests).

Mixed-effects multilevel meta-regression models were also fitted using REML in *metafor* (*rma.mv*), with effect sizes nested within studies and t-distribution-based inference. For continuous exploratory moderators (e.g., CM dosage), linear, quadratic, and cubic specifications were compared, with corrected Akaike information criterion (AICc) guiding parsimonious model selection [43,44]. Nonlinear forms were retained only when they provided materially better fit and were supported by data coverage.

All visualizations were produced using *ggplot2* and *orchaRd* [45]. Given the number of moderator tests conducted and the absence of formal adjustment for multiple testing, subgroup and meta-regression analyses were considered exploratory, particularly for categories with sparse data. Accordingly, interpretation focused on between-subgroup comparisons and sensitivity analyses rather than isolated statistically significant within-subgroup findings.

#### 2.5.4. Risk of Publication Bias and Sensitivity Analyses

The contour-enhanced funnel plot [46], along with Egger’s asymmetry test [47,48], was employed to assess publication bias (tests were only conducted when k ≥ 10) [49], with *p* > 0.05 indicating no risk of publication bias. Funnel plot symmetry was evaluated through both visual inspection and Egger’s test, allowing for a combined subjective and statistical assessment of potential small-study effects and publication bias.

Sensitivity analyses were conducted within the three-level meta-analytic framework to evaluate the robustness of the main findings. First, we examined the impact of varying the assumed correlation coefficient used in standard error estimation. Second, leave-one-out analyses were performed by systematically excluding each study to assess its influence on the pooled effect size. Third, outlier and influential case diagnostics were performed using model-based statistics, including Cook’s distance, Hat value [50] and studentized residuals [51], at both the within-study level (Level 2) and between-study level (Level 3). Observations were considered potentially influential if their Hat values or Cook’s distances exceeded three times the respective mean values, or if the absolute value of their studentized residuals was greater than 3. The meta-analytic model was re-estimated with these outliers excluded to determine the extent to which they influenced the overall conclusions.

### 2.6. Certainty of the Evidence

The Grading of Recommendations Assessment, Development, and Evaluation (GRADE) framework was applied to rate certainty of evidence as high, moderate, low, or very low, considering risk of bias, and inconsistency, indirectness, imprecision, and publication bias [52]. Domain-specific criteria used for downgrading are provided in Supplementary Material S4. For subgroup analyses, sparse evidence was considered primarily within the imprecision domain, and certainty ratings for sparse subgroups were interpreted cautiously and mainly for descriptive purposes. Assessments were independently performed by one reviewer and cross-verified by a second reviewer to ensure accuracy and consistency.

## 3. Results

### 3.1. Studies Retrieved

**Figure 1:**
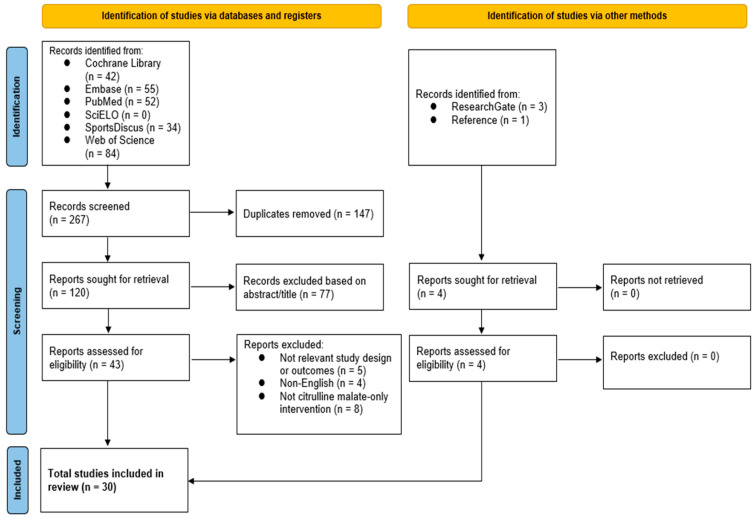
PRISMA flow diagram for included and excluded studies.

The initial search yielded 271 publications: 267 from the primary database search, 4 obtained from other sources. After screening, 30 studies were deemed eligible to be included in the meta-analysis (Figure 1).

### 3.2. Characterization of Participants

Across all studies, sample sizes ranged from 10 to 64 participants, for a total of 644 participants. Of these 644 participants, 504 were male and 140 were female. Most of the included studies recruited male-only participants (18 studies; effect sizes k = 91), a few studies recruited both males and females (9 studies; k = 31), and only three studies recruited female-only participants (k = 16). In addition, most studies recruited trained participants (21 studies; k = 109), while a few studies recruited untrained participants (9 studies; k = 29). For more details, please refer to Table 1.

#### 3.2.1. Exercise Type and Perceived Exertion

According to the type of exercise categorized by the primary underlying energy system or neuromuscular mechanism, six studies assessed aerobic endurance (k = 16), six studies evaluated anaerobic performance (k = 21), fifteen studies examined strength/power (k = 48), and twenty studies investigated muscular endurance (k = 53). Additionally, perceived exertion outcomes were reported in eight studies (k = 19). For more details, please refer to Table 1.

#### 3.2.2. Supplementation Protocol and Study Design

CM supplementation was provided in solid form in 4 studies (k = 10) and in beverage form in 26 studies (k = 128). With the exception of Naimah et al. (2022) [69], who applied weight-adjusted dosing, all trials used fixed doses, most commonly 8 g and, less frequently, 12 g. Acute single doses were administered 40–120 min before exercise, whereas repeated supplementation protocols ranged from 3 to 56 days. All studies employed double-blind designs, including 24 randomized cross-over and 6 randomized parallel-group trials (Table 1).

### 3.3. Primary Analysis

Across studies, CM was associated with a small, statistically significant improvement in overall exercise performance (Hedges’ g = 0.16, 95% CI [0.05, 0.27]; *p* = 0.01; k = 138; Low GRADE), with moderate heterogeneity (*I*^2^ = 45%) and low statistical power (8%); the prediction interval was −0.37 to 0.69. When stratified by intake duration, acute single dose supplementation showed a small, statistically significant benefit (g = 0.16, 95% CI [0.02, 0.30]; *p* = 0.02; k = 114; *I*^2^ = 52%; power = 9%; Low GRADE), whereas chronic supplementation yielded a small effect that did not reach significance (g = 0.13, 95% CI [−0.01, 0.28]; *p* = 0.08; k = 24; *I*^2^ = 0%; power = 7%; Very low GRADE).

**Figure 2:**
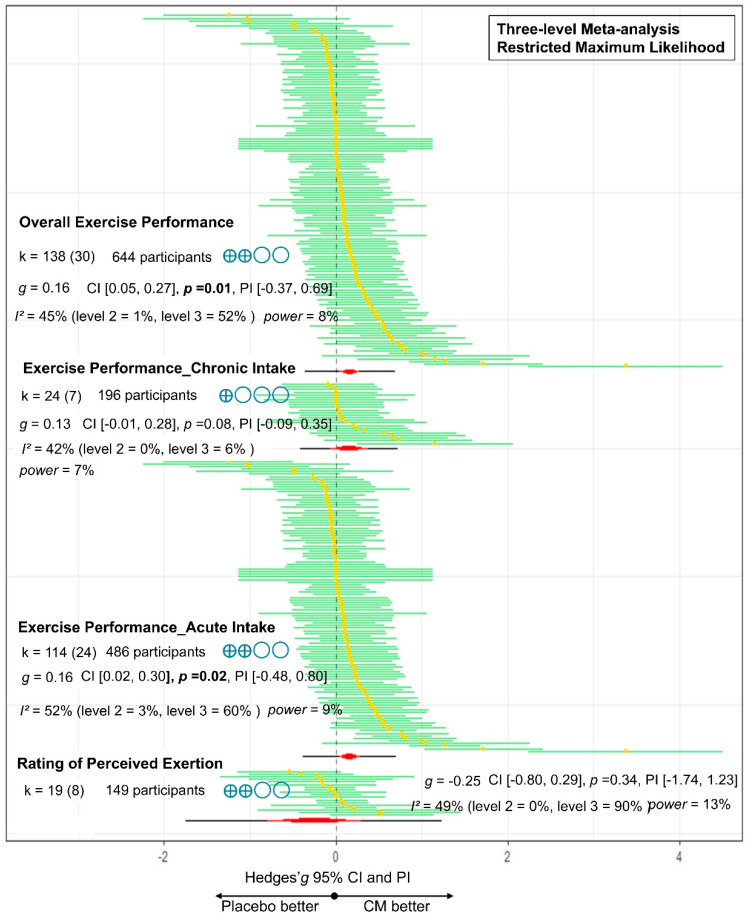
Primary pooled effect sizes of citrulline malate supplementation on exercise performance (acute/chronic) and perceived exertion. Notes: K, the total number of effects included in the pooled effect size; Hedges’ g, the effect size indicators used in the pooled; 95%CI, 95% confidence interval; PI, prediction Interval; *p*-value, statistically significant *p* values for pooled results; *I*^2^, quantitative indicators of heterogeneity; Power, exploratory post hoc statistical power estimate for pooled effect size; Blue Circles, GRADE, grading of recommendations assessment, development, and evaluation, a system for evaluating the quality of evidence and strength of recommendations.

For perceptual outcomes, the pooled effect favored lower subjective fatigue/exertion scores, but was not statistically significant (g = −0.25, 95% CI [−0.80, 0.29]; *p* = 0.34; k = 19; *I*^2^ = 49%; power = 13%; Low GRADE; PI [−1.74, 1.23]). For more details, please refer to Figure 2, and also see Supplementary Material S5 for a traditional forest plot with study labels and weights.

### 3.4. Moderator Analysis

**Figure 3:**
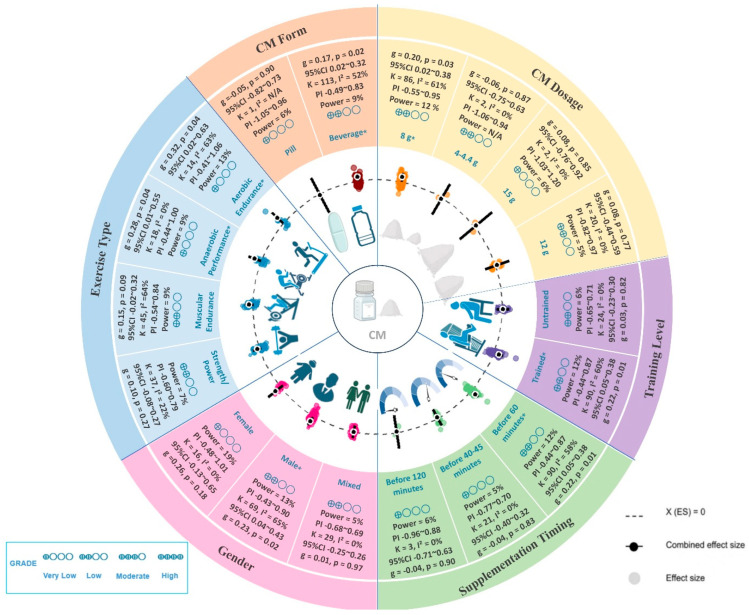
Moderator analyses of primary outcomes under acute supplementation. Notes: CM, Citrulline malate; K, the total number of effects included in the pooled effect size; ES, Hedges’ g, the effect size indicators used in the pooled; 95%CI, 95% confidence interval; PI, prediction Interval; *p*-value, statistically significant *p* values for pooled results; *I*^2^, quantitative indicators of heterogeneity; Power, exploratory post hoc statistical power estimate for pooled effect size; Blue Circles, GRADE, grading of recommendations assessment, development, and evaluation, a system for evaluating the quality of evidence and strength of recommendations; *, Represents statistical significance, *p* < 0.05.

In exploratory subgroup and meta-regression analyses of acute CM trials, we evaluated sex, training status, exercise category, CM form, CM dosage and supplementation timing. Detailed numerical subgroup results corresponding to Figure 3 are provided in Supplementary Material S6.

#### 3.4.1. Potential Moderators of Participant Characterization

By sex, the within-subgroup estimate was statistically significant in males (g = 0.23, 95% CI [0.04, 0.43]; *p* = 0.02; k = 69; PI [−0.43, 0.90]; *I*^2^ = 65%), non-significant in females (g = 0.26, 95% CI [−0.13–0.65]; *p* = 0.18; k = 16), and null in mixed samples (g = 0.01, 95% CI [−0.25, 0.26]; *p* = 0.97; k = 29). The between-subgroup test was not significant (*p_between* = 0.32) (Figure 3).

By training status, the within-subgroup estimate was statistically significant in trained participants (g = 0.22, 95% CI [0.05, 0.38]; *p* = 0.01; k = 90; PI [−0.44, 0.87]; *I*^2^ = 60%), whereas the estimate in untrained participants was not significant (g = 0.03, 95% CI [−0.23, 0.30]; *p* = 0.82; k = 24). The subgroup difference was not significant (*p_between* = 0.25) (Figure 3).

#### 3.4.2. Potential Moderators of Exercise Type

Across exercise categories, within-subgroup estimates were statistically significant for aerobic endurance (g = 0.32, 95% CI [0.02, 0.63]; *p* = 0.04; k = 14; PI [−0.41, 1.06]; *I*^2^ = 63%) and anaerobic performance (g = 0.28, 95% CI [0.01, 0.55]; *p* = 0.04; k = 18; PI [−0.44, 1.00]; *I*^2^ = 9%), whereas estimates for muscular endurance (g = 0.15, 95% CI [−0.02–0.32]; *p* = 0.09; k = 45; *I*^2^ = 64%) and strength/power (g = 0.10, 95% CI [−0.08, 0.27]; *p* = 0.28; k = 37; *I*^2^ = 22%) were not significant. The overall between-category test was not significant (*p_between* = 0.48) (Figure 3).

#### 3.4.3. Potential Moderators of Supplementation Protocol

For supplementation timing, the within-subgroup estimate for ~60 min pre-exercise dosing was statistically significant (g = 0.22, 95% CI [0.05, 0.38]; *p* = 0.01; k = 90; PI [−0.44, 0.87]; *I*^2^ = 58%), whereas estimates for ~40–45 min (g = −0.04; *p* = 0.83; k = 21) and ~120 min (g = −0.04; *p* = 0.90; k = 3) were not significant. Between-subgroup differences were not significant (*p_between* = 0.37) (Figure 3).

**Figure 4:**
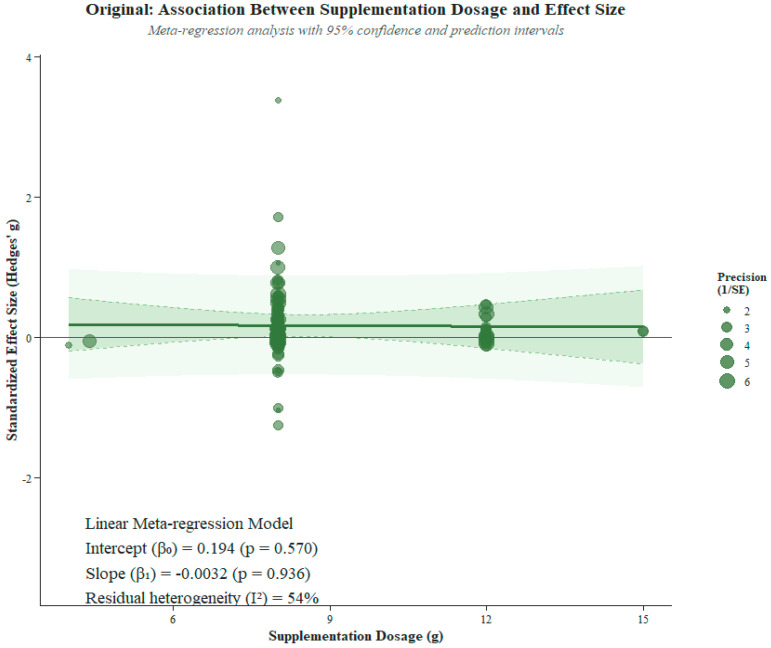
Regression analysis on the dosage of acute single-dose citrulline malate supplementation. Notes: β0 represents the intercept; β1 represenst the slopes; *p*-value, statistically significant *p* values for pooled results; *I*^2^ means heterogeneity; The green shaded part represents the 95% confidence interval; The outermost green dotted line represents the prediction interval.

By dosage category, the within-subgroup estimate for 8 g was statistically significant (g = 0.20, 95% CI [0.02, 0.38]; *p* = 0.03; k = 86; PI [−0.55, 0.95]; *I*^2^ = 61%), whereas estimates for 4–4.4 g (k = 2), 12 g (g = 0.08, 95% CI [−0.44, 0.59]; *p* = 0.77; k = 56; *I*^2^ = 5%), and 15 g (k = 2) were not significant. The test for subgroup differences was not significant (*p_between* = 0.87) (Figure 3). Consistently, exploratory linear meta-regression with total CM dose showed no dose–response association (β_1_ = −0.003, *p* = 0.94; residual *I*^2^ = 54%) (Figure 4).

Finally, by formulation, the within-subgroup estimate for beverage trials was statistically significant (g = 0.17, 95% CI [0.02, 0.32]; *p* = 0.02; k = 113; PI [−0.49, 0.83]; *I*^2^ = 52%), while the pill subgroup (k = 1) was inconclusive. The between-formulation comparison was not significant (*p_between* = 0.58) (Figure 3).

### 3.5. Risk of Bias and Quality of Methods

The risk of bias for each study is depicted in Supplementary Material S7, and detailed RoB 2 assessment records are available in the OSF project (osf.io/tez3a). Of the included trials, the large majority (approximately 80–85%) were rated as having an overall risk of bias of “some concerns,” with only a small fraction judged as low risk and a minority classified as high risk. The most frequent source of bias arose from the randomization process, where inadequate reporting of sequence generation and allocation concealment was common. Among crossover trials, washout periods were generally considered adequate, and bias arising from period and carryover effects was usually rated as low risk, with some concerns in a small number of studies. In contrast, most studies were rated as low risk for deviations from intended interventions and missing outcome data. The domains of outcome measurement and selective reporting were most often judged as some concerns, with occasional high risk due to incomplete specification of prespecified outcomes. Overall, while outcome collection and follow-up were generally sound, trial design and reporting weaknesses, particularly in randomization and selective reporting, should be considered when interpreting the pooled results.

Funnel plots and Egger’s regression were applied to categories with at least 10 effect sizes. Potential small-study effects were detected for overall exercise performance and for CM supplementation analyzed as acute and as chronic. Within the acute trials, Egger’s test indicated bias in the subgroups of male samples, trained participants, ingestion approximately 60 min before exercise, 8 g and 12 g doses, and beverage formulations. No clear evidence of funnel-plot asymmetry or small-study effects was detected in the remaining categories (*p* > 0.05; Supplementary Material S8). Across categories, statistical power was generally low (Supplementary Material S9).

The mean PEDro score for all studies was 7.4, indicating that the methodological quality of the included studies was generally good (Supplementary Material S10). In addition, certainty ratings across outcomes ranged from low to very low, reflecting limitations related to risk of bias, inconsistency, imprecision, and/or publication bias. Detailed GRADE assessments are provided in Supplementary Material S4.

### 3.6. Sensitivity Analysis

We conducted sensitivity analyses to assess robustness. Using an alternative within-study correlation of r = 0.80 for the chronic supplementation subset yielded a small statistically significant effect (g = 0.15; 95% CI 0.01 to 0.30; *p* = 0.047). In contrast, after outlier removal, the effect of perceived exertion changed direction and was no longer significant (g = 0.01; 95% CI −0.10 to 0.12; *p* = 0.87) (Supplementary Material S11). Leave-one-out analyses further highlighted instability: for acute single-dose supplementation, exclusion of Pérez-Guisado and Jakeman (2010) rendered the pooled effect non-significant (g = 0.10; *p* = 0.06; *I*^2^ = 0%) [5], while for perceived exertion, exclusion of Glenn et al. (2017) substantially attenuated the effect estimate (g = −0.02; *p* = 0.85; *I*^2^ = 0%) (Supplementary Material S12) [61]. Results for other primary comparisons were materially unchanged.

In acute-trial subgroup analyses with outliers excluded, the effect for male samples attenuated but remained significant (g = 0.12; 95% CI 0.01 to 0.26; *p* = 0.046), whereas effects for aerobic endurance (g = 0.09; 95% CI −0.20 to 0.38; *p* = 0.56) and anaerobic performance (g = 0.01; 95% CI −0.26 to 0.28; *p* = 0.96) were reduced and no longer significant. The timing category at 40–45 min before exercise, the 4–4.4 g and 15 g dose categories, and the pill formulation were entirely excluded as outliers (Supplementary Material S13). Finally, linear meta-regression of total CM dose remained non-significant after outlier exclusion, indicating no evidence of a dose–response relationship (Supplementary Material S14).

Overall, these sensitivity analyses suggest that several apparently significant findings, particularly for acute supplementation, perceived exertion, aerobic endurance, and anaerobic performance, should be interpreted cautiously.

## 4. Discussion

This meta-analysis synthesizes current evidence on CM supplementation and exercise performance across a range of exercise domains. Overall, CM supplementation was associated with small improvements in performance outcomes, whereas effects on perceived exertion were inconsistent and not statistically significant. Pooled analyses suggested that evidence for ergogenic effects appeared somewhat more consistent in acute supplementation protocols, while findings for chronic supplementation remain limited and uncertain. However, the magnitude of the pooled effects was small, certainty of evidence was low to very low, and sensitivity analyses indicated that several findings were not robust. These considerations should temper interpretation of the results and suggest that any potential benefits of CM are likely to be modest and context-dependent.

### 4.1. Acute vs. Chronic

While acute CM supplementation was associated with small improvements in pooled analyses, evidence for chronic supplementation remains limited and uncertain. This pattern should be interpreted cautiously because the number of chronic trials was small and their designs varied in duration, dosage, and outcomes. One hypothesis is that sustained citrulline exposure could induce physiological adaptations that attenuate the acute ergogenic response. For example, prolonged high arginine availability could, in theory, influence arginase activity [75], endogenous nitric oxide synthase (NOS) inhibitors such as asymmetric dimethylarginine (ADMA) [76], or (endothelial nitric oxide synthase) eNOS coupling [77], thereby modifying the translation of elevated citrulline availability into nitric oxide-related effects. In addition, a metabolic ceiling in urea-cycle activity or lactate/ammonia clearance during longer-term supplementation could plausibly contribute to diminishing marginal returns [5,6]. Although longer-term CM intake may increase circulating nitric oxide metabolite (NOx) levels [78,79], sustained elevations in biochemical markers do not necessarily translate into improved exercise performance. Similarly, malate could theoretically support oxidative metabolism through anaplerotic entry into the TCA cycle [2], but any such effect may be transient or masked in longer supplementation protocols. These mechanisms remain biologically plausible but speculative in the context of the present meta-analysis and require direct testing in future studies.

### 4.2. Acute Supplementation

#### 4.2.1. Characterization of Participants

Sex-specific findings were mixed and remain limited by the small number of female-only studies. Small but significant within-subgroup benefits were observed in male samples, whereas studies involving female or mixed cohorts did not demonstrate clear improvements, and the male effect attenuated after outlier removal although it remained statistically significant. Although not directly confirmed, several biological mechanisms may help explain these exploratory patterns. Sex hormones, particularly estrogen, have well-documented effects on eNOS activity and vascular reactivity, leading to sex-based variability in NO-mediated vasodilation [80,81]. In the three included trials that recruited female participants [59,60,61], control of menstrual-cycle fluctuations and the use of hormonal contraceptives differed across studies, which may have further contributed to inconsistent outcomes [82]. Differences in arginine metabolism, including arginase activity and endogenous NOS inhibition, may also be relevant, along with sex differences in muscle mass and absolute workload [83]. The near-zero non-significant effect observed in mixed-sex cohorts further highlights the uncertainty regarding potential benefits among female participants.

Findings by training status were also inconclusive. A small but significant within-subgroup effect was observed in trained participants, whereas the estimate in untrained individuals was not significant; however, the between-subgroup test was not significant. Regular training enhances skeletal-muscle capillarization, endothelial responsiveness, and mitochondrial efficiency [84,85]. These adaptations could theoretically affect CM-related changes in perfusion and metabolite clearance during high-intensity exercise. By contrast, lower vascular reactivity and higher performance variability in untrained cohorts could attenuate or obscure small acute effects.

Taken together, sex and training status may be useful factors to consider in future research, but the present subgroup analyses do not provide clear evidence of differential CM effects. However, these interpretations should be considered cautiously because female-only samples were sparse, heterogeneity across protocols was evident, and statistical power across subgroups was limited.

#### 4.2.2. Exercise Type and Perceived Exertion

Across exercise modalities, statistically significant within-subgroup effects were observed in aerobic endurance and anaerobic performance before outlier control, but both effects were attenuated and became non-significant after outlier removal. Muscular endurance retained a very small, marginal effect both before and after outlier exclusion, consistent with prior evidence for modest increases in repetitions to failure with acute CM [15]. In contrast, strength and power outcomes remained trivial both before and after outlier control, which is consistent with previous meta-analytic findings indicating that CM does not clearly enhance maximal strength in resistance-trained adults [16].

This pattern is broadly consistent with physiological expectations. Transient increases in arginine availability could theoretically augment nitric oxide-mediated vasodilation and hasten the adjustment of oxygen uptake during severe-intensity work, which may be relevant to efforts lasting 10 to 60 s that depend substantially on anaerobic glycolysis and to longer aerobic tasks requiring oxidative support and metabolite clearance [2,78,86]. Malate, as a tricarboxylic acid cycle intermediate, could in theory support oxidative ATP synthesis and thereby favor endurance-type outcomes [2], although direct evidence for an independent contribution of malate is lacking and narrative reviews emphasize the current uncertainty [11,17]. By contrast, maximal force generation in efforts of ≤10 s is determined predominantly by neuromuscular factors and phosphocreatine availability, processes that may be less responsive to short-term CM. Taken together with signs of publication bias in several acute subgroups and the loss of significance after outlier removal, these results suggest that any modality-specific ergogenic effects are small, uncertain, and hypothesis-generating.

With respect to perceived exertion and fatigue, pooled analyses suggested a tendency toward lower subjective fatigue, but this effect was not statistically significant and was highly unstable across sensitivity models. Potential explanations remain speculative. CM could theoretically influence peripheral metabolic efficiency through nitric oxide-mediated perfusion, ammonia clearance, or malate-supported anaplerosis, which might allow greater absolute work to be performed at a similar subjective effort [2,5,78]. In addition, many included trials used self-paced or time-to-exhaustion protocols in which end-test perceived exertion may approach a ceiling, limiting the capacity to detect perceptual changes [53,61,63]. Variation in perceptual scales, timing of measurement, and reporting practices may further dilute potential effects. Thus, the pattern of “improved performance without consistent reductions in perceived exertion or fatigue” should be interpreted cautiously and highlights the need for more standardized perceptual assessments in future CM trials.

### 4.3. Supplementation Protocol

There was limited diversity in the timing of CM ingestion, with most studies opting for intake 1 h before exercise [87], which restricted our ability to examine a full time–concentration–effect relationship. In subgroup analyses, a small within-subgroup effect was observed at this interval, whereas earlier or later ingestion windows showed no clear effects; however, between-subgroup differences were not significant. This pattern is biologically plausible in light of citrulline pharmacokinetics. In a dose-ranging study, oral L-citrulline at 2, 5, 10, and 15 g achieved peak plasma concentrations at approximately 1 h, followed by a rapid decline within 15–30 min [88]. Very few performance trials have concurrently profiled circulating amino acids. One included study administering 12 g CM reported that plasma citrulline rose to 343 ± 41 μM at 60 min compared with 39 ± 12 μM after placebo, with a similar increase in plasma ornithine (9.5 ± 3.1 μM vs. 2.4 ± 1.6 μM) [53]. Because only a single blood sample was obtained at 60 min, the temporal dynamics of these metabolites relative to exercise remain unresolved. Notably, the independent pharmacokinetic contribution of malate is still uncertain, although as a tricarboxylic acid-cycle intermediate it could theoretically influence absorption and downstream bioavailability.

With respect to dose, most acute trials used 8 g CM. In our moderator analyses, a small within-subgroup effect was observed at the commonly used 8 g dose, whereas lower doses of 4–4.4 g and higher doses of 12 g and 15 g did not show clear effects, and subgroup contrasts were not significant. Linear dose–response was not detected in meta-regression and remained null after outlier exclusion. By comparison, pharmacokinetic data from pure L-citrulline show graded increases in plasma levels from 2 to 15 g, with a peak at 15 g that was significantly higher than 10 g, suggesting that larger intakes, potentially exceeding 10 g, may be necessary to maximize ergogenic effects under some conditions, a possibility not yet verified for CM [17,88]. From a practical perspective, dietary sources cannot reliably deliver such quantities of citrulline at the time needed for performance. Approximately 3–5 kg of fresh watermelon would be required to achieve a citrulline intake of 10 g [89]. Similarly, although malate occurs naturally in many fruits, typical dietary intake does not provide the concentrated amounts or prescribed citrulline–malate ratios used in supplementation trials [17].

Given the relatively large doses used in trials assessing possible ergogenic effects, supplementation is typically achieved through commercial supplements or the raw chemical compound. Almost all acute studies used CM as a beverage, and this subgroup showed a small statistically significant within-subgroup effect. However, the between-formulation comparison was not significant. Only one crossover trial of 4.4 g capsules failed to improve exercise performance, so the capsule data are insufficient to draw conclusions [54].

Beyond dosage form, product composition complicates inference. While malate may theoretically contribute to exercise performance through its role in aerobic metabolism, the primary rationale for its inclusion in CM formulations likely relates to pharmaceutical considerations rather than ergogenic effects. Specifically, malate serves to form a stable salt complex with citrulline for improved storage stability and to neutralize the inherent alkalinity of citrulline, facilitating palatability and gastric tolerance [11]. This formulation approach, while practical for supplement manufacturing, may inadvertently dilute the active citrulline content in commercial products.

Regarding the ratio of citrulline to malate in beverages, most research studies report using a 2:1 citrulline:malate ratio. Independent assessments indicate that commercial products labeled as 2:1 citrulline-to-malate often deviate toward lower ratios, sometimes near 1.1:1, which would reduce the delivered citrulline for a given serving and could attenuate performance effects [11]. Given that any ergogenic benefit of CM likely depends primarily on adequate citrulline exposure, verified dosing and transparent product specifications are essential in both research and practice [87].

However, recent evidence challenges the overall efficacy of both formulations. A well-controlled crossover study comparing pure L-citrulline (5.3 g) versus citrulline–malate (5.3 g citrulline + 2.7 g malate) found that neither supplement improved maximal neuromuscular performance, ballistic strength, or strength-endurance during resistance training in young, trained adults (all *p* > 0.061) [67]. These null findings, combined with the uncertain additive value of malate, suggest that future research should critically examine whether the modest effects reported in earlier studies translate to meaningful performance improvements in well-trained populations.

Together, these findings underscore the need for studies that integrate pharmacokinetic sampling with standardized performance testing, while also clarifying whether the complexity of combination formulations is justified given the limited and inconsistent evidence base.

### 4.4. Future Directions

Several avenues should be prioritized to resolve the remaining uncertainties surrounding CM and to improve the clinical and applied relevance of future findings. First, adequately powered, preregistered randomized trials are needed across diverse populations. The current evidence base is dominated by small acute studies in young men. Future work should recruit larger female cohorts with explicit control of menstrual cycle phase and contraceptive use, and should include older adults, adolescents, and athletes at different training levels. Stratified or covariate-adjusted analyses by sex and training status should be planned a priori.

Second, the effects of chronic CM supplementation require dedicated evaluation. Our analyses did not identify clear benefits with chronic use, which raises questions about potential counter-regulation and adherence. Future trials should test multiweek and multimonth protocols with standardized outcomes, serial pharmacokinetic and mechanistic sampling, predefined safety reporting, and designs that can detect desensitization, carryover, or periodized use benefits.

Third, supplementation regimens require systematic optimization. Dose-ranging CM trials that include and exceed the commonly used 8 g dose are warranted, with concurrent safety monitoring and standardized adverse-event reporting. Timing should be tested beyond the prevailing 60-min window, using pharmacokinetic and pharmacodynamic sampling before and after exercise to establish a time–concentration–effect relationship. Head-to-head comparisons of CM versus pure L-citrulline, matched for delivered citrulline, will help clarify whether malate confers any independent or synergistic benefit. Parallel comparisons of beverage and capsule formulations, as well as single versus split dosing, are also needed.

Fourth, mechanistic endpoints should be integrated with performance outcomes. Studies should measure circulating citrulline, arginine, ornithine, nitric-oxide-related markers, and indices of ammonia and lactate handling during standardized exercise tasks. Noninvasive physiology, including near-infrared spectroscopy, Doppler measures of limb blood flow, and if feasible ^31P-MRS, can link potential perfusion and metabolic effects to task-specific performance across domains. The independent pharmacokinetic contribution of malate remains uncertain and requires targeted investigation.

Fifth, product verification and reporting must be strengthened. Investigators should independently assay the citrulline to malate ratio and absolute citrulline content of the tested supplement and include certificates of analysis as Supplementary Material. Transparent reporting of brand, batch, formulation, and storage conditions will improve reproducibility and reduce misclassification bias.

Sixth, real-world relevance and integration. Future work should evaluate whether CM meaningfully improves competitive performance in applied contexts, including endurance races, intermittent team-sport scenarios, and high-stakes competition settings, using adequately powered field studies with sport-specific outcomes. Potential interactions with common ergogenic aids such as β-alanine, dietary nitrate, and caffeine remain largely unexplored and warrant factorial or crossover trials that are carefully matched for delivered citrulline dose and timing.

### 4.5. Strength and Limitations

This is the first systematic review and meta-analysis to comprehensively synthesize evidence on CM supplementation across multiple exercise domains, including strength, anaerobic performance, muscular endurance, aerobic endurance, and perceived exertion. A broad literature search was conducted across six databases with independent screening and data extraction by two reviewers, thereby minimizing the risk of selection bias. Methodologically, we employed a three-level meta-analytic framework to appropriately account for the dependency of multiple outcomes within individual studies and to avoid statistical double counting. The stability of results was tested through sensitivity analyses, including alternative correlation coefficients, exclusion of outliers, and model adjustments, enhancing the robustness of our conclusions. Furthermore, we applied the GRADE approach to transparently evaluate the certainty of evidence for each outcome, which improves interpretability and utility for researchers and practitioners.

Despite these strengths, several limitations should be acknowledged. First, although exercise outcomes were grouped according to their dominant energetic system or neuromuscular mechanism to improve interpretability and reduce model instability, many exercise protocols involve overlapping metabolic demands. Therefore, distinctions between strength/power, anaerobic performance, muscular endurance, and aerobic endurance should be viewed as pragmatic classifications rather than strict physiological boundaries. Second, comparisons between acute and chronic supplementation protocols should also be interpreted cautiously because these studies differed not only in supplementation duration but also in participant training background, possible adaptation effects, study quality, and testing procedures. More broadly, substantial heterogeneity was observed in several analyses, likely reflecting differences in study populations, supplementation protocols, dosing regimens, and outcome measures. In particular, inconsistent reporting and limited independent verification of citrulline-to-malate ratios or absolute citrulline content may have contributed to exposure misclassification. Third, evidence of publication bias was detected in several acute subgroups, suggesting that small-study effects may have inflated certain estimates. Fourth, many included trials were limited by small sample sizes and incomplete reporting of randomization procedures, which collectively reduce the certainty of the findings. Finally, post hoc statistical power remained low across most outcomes, restricting the ability to detect modest but practically relevant effects. Taken together, these limitations indicate that current evidence should be interpreted cautiously when translating findings into practical recommendations. They also underscore the need for larger, preregistered, and methodologically rigorous trials to confirm and extend the present findings.

### 4.6. Practical Applications

CM supplementation may produce small, uncertain, and context-dependent effects on exercise performance. Exploratory findings suggest that potential benefits may be more apparent in some acute endurance or anaerobic tasks, but these observations were not robust across sensitivity analyses and require confirmation. Most studies used acute protocols of approximately 8 g administered around 60 min before exercise; however, current evidence does not establish a reliable dosing strategy, ingestion timing, formulation, or target population. Athletes and practitioners should therefore interpret the potential ergogenic effects of CM cautiously and avoid assuming consistent performance benefits across individuals or exercise settings.

## 5. Conclusions

This systematic review and three-level meta-analysis suggests that CM supplementation may be associated with small improvements in exercise performance, but certainty of evidence remains low to very low and prediction intervals were wide. Evidence appeared somewhat more consistent for acute than chronic supplementation, although interpretation is limited by small effect sizes, potential publication bias or small-study effects, and a lack of robust subgroup evidence. Larger, well-controlled trials with verified supplement composition and standardized protocols are needed to clarify the magnitude, consistency, and practical relevance of any potential ergogenic effects.

## Figures and Tables

**Table 1:** Summary characteristics and reported findings of included studies.

Study	Design	Participants	CM Protocol	Exercise Tests/Outcomes	Summary of Reported Findings
Bayat et al. (2025) [7]	Parallel RCT	22 resistance-trained males	12 g CM, beverage, 42 days + 60 min pre-test	Hack squat, bench press, leg extension, incline press	Improved bench press repetitions only; no RPE effect
Bezuglov et al. (2022) [10]	Parallel RCT	18 elite male soccer players	3 g or 6 g CM, beverage, 45 min pre-test	Football-specific sprint/dribbling test	No significant performance or RPE effect
Chappell et al. (2018) [11]	Crossover RCT	15 moderately trained adults	8 g CM, beverage, 60 min pre-test	Barbell curls; quadriceps strength	No significant performance effect
Chappell et al. (2020) [12]	Crossover RCT	19 moderately trained adults	8 g CM, beverage, 60 min pre-test	Barbell curls	No significant performance effect
Chappell et al. (2024) [8]	Crossover RCT	11 resistance-trained male rugby players	8 g/day CM, beverage, 3 days + 60 min pre-test	Barbell curls; quadriceps strength	Improved barbell curl repetitions only
Cunniffe et al. (2016) [53]	Crossover RCT	11 well-trained males	12 g CM, beverage, 60 min pre-test	Repeated cycling sprints; cycling to exhaustion	Improved first-sprint mean power only; no RPE effect
Devrim-Lanpir et al. (2024) [54]	Crossover RCT	21 recreationally active males	4.4 g CM, pill, 60 min pre-test	CrossFit workout rounds	No significant performance effect
Faria and Egan (2024) [55]	Crossover RCT	13 male team-sport athletes	8 g/day CM, tablets, 3 days + 180 min pre-test	Repeated 40 m sprints	Improved fastest sprint time only
Farney et al. (2019) [56]	Crossover RCT	12 recreationally active adults	8 g CM, beverage, 60 min pre-test	Isokinetic leg extension; high-intensity exercise circuit	No significant performance effect
Fick et al. (2021) [57]	Crossover RCT	18 recreationally active males	8 g CM, beverage, acute + 7 days	Maximal leg extensions	No significant performance effect
Gills et al. (2021) [58]	Crossover RCT	28 recreationally active males	8 g CM, beverage, 60 min pre-test	Cycling TTE; Wingate test	No significant performance effect
Gills et al. (2023) [59]	Crossover RCT	19 recreationally active females	8 g CM, beverage, 60 min pre-test	Isokinetic leg extension	No significant performance effect
Glenn et al. (2016) [60]	Crossover RCT	17 female master tennis athletes	8 g CM, beverage, 60 min pre-test	Grip strength; vertical jump; Wingate test	Improved grip strength and Wingate outcomes
Glenn et al. (2017) [61]	Crossover RCT	15 resistance-trained females	8 g CM, beverage, 60 min pre-test	Bench press and leg press to exhaustion	Improved repetitions to exhaustion and reduced RPE
Gonzalez et al. (2018) [62]	Crossover RCT	12 resistance-trained males	8 g CM, beverage, 40 min pre-test	Bench press repetitions and power	No significant performance effect
Grala et al. (2021) [63]	Parallel RCT	18 male university students	6 g CM, beverage, 7 days	Leg extension to exhaustion; treadmill test	No significant performance or RPE effect
Haugen et al. (2023) [64]	Crossover RCT	35 resistance-trained adults	12 g CM, beverage, 60 min pre-test	Countermovement jump; 1RM squat/bench press; repetitions to exhaustion	No significant performance effect
Hwang et al. (2018) [65]	Parallel RCT	50 resistance-trained males	2 g/day CM, capsule, 28 and 56 days	1RM bench press and leg press	No significant performance effect
Jafari et al. (2024) [66]	Crossover RCT	12 professional male wrestlers	8 g CM, beverage, 60 min pre-test	Burpee test; grip strength; back-leg-chest strength; wrestling skills	Improved selected strength and wrestling-specific outcomes only; no RPE effect
Martín-Olmedo et al. (2024) [67]	Crossover RCT	43 resistance-trained adults	8 g CM, beverage, 45 min pre-test	Squat/bench press; countermovement jump; bench press throw; repetitions to exhaustion	No significant performance or RPE effect
Mayo et al. (2023) [68]	Parallel RCT	32 recreationally active adults	4 g or 8 g CM, beverage, 45 min pre-test	300-yard shuttle run	No significant performance effect
Naimah et al. (2022) [69]	Crossover RCT	12 trained judokas	250 mg/kg CM, beverage, 60 min pre-test	Wingate test; grip strength	Improved peak power and average grip strength only
Newbury et al. (2024) [70]	Crossover RCT	11 highly trained swimmers	15 g CM, beverage, 60 min pre-test	Repeated 300 m swimming	No significant performance effect
Pérez-Guisado and Jakeman (2010) [5]	Crossover RCT	41 trained males	8 g CM, beverage, 60 min pre-test	Bench press repetitions	Improved most bench press repetition outcomes
Sharma et al. (2014) [9]	Crossover RCT	20 male collegiate athletes	8 g CM, beverage, 60 min pre-test	Vertical jump; graded cycling test	Improved vertical jump and VO_2_max-related fatigue response
Tishchenko et al. (2023) [71]	Parallel RCT	64 national-level athletes	8 g/day CM, capsule, 30 days	Submaximal cycling power	Original statistical significance not clearly reported
Trexler et al. (2019) [1]	Crossover RCT	27 recreationally active males	8 g CM, beverage, 120 min pre-test	Maximal-effort leg extension	No significant performance effect
Viana et al. (2021) [72]	Crossover RCT	10 male judo athletes	8 g CM, beverage, 60 min pre-test	Special Judo Fitness Test	No significant performance or RPE effect
Wax et al. (2015) [73]	Crossover RCT	12 advanced resistance-trained males	8 g CM, beverage, 60 min pre-test	Leg press, hack squat, leg extension to exhaustion	Improved repetitions across resistance exercises
Wax et al. (2016) [74]	Crossover RCT	14 resistance-trained males	8 g CM, beverage, 60 min pre-test	Chin-ups, reverse chin-ups, push-ups	Improved total repetitions

Table notes: This table provides a concise summary of study characteristics and originally reported findings. Detailed numerical extraction data and effect-size calculation details are provided in the OSF dataset at osf.io/tez3a. CM, citrulline malate; g, grams; min, minutes; RCT, randomized controlled trial; RPE, rating of perceived exertion; TTE, time to exhaustion; 1RM, one-repetition maximum.

## Data Availability

All Data Analyzed in this Study were Obtained from Previously Published Studies, Which are Cited in the Manuscript. No New Data were Generated for this Study.

## Supplementary Materials

The following supporting information can be downloaded at: https://www.mdpi.com/article/10.3390/nu18121881/s1.

## References

[1] Trexler E.T., Keith D.S., Schwartz T.A., Ryan E.D., Stoner L., Persky A.M., Smith-Ryan A.E. (2019). Effects of Citrulline Malate and Beetroot Juice Supplementation on Blood Flow, Energy Metabolism, and Performance during Maximum Effort Leg Extension Exercise. J. Strength Cond. Res..

[2] Bendahan D., Mattei J.P., Ghattas B., Confort-Gouny S., Guern M.E.L., Cozzone P.J. (2002). Citrulline/Malate Promotes Aerobic Energy Production in Human Exercising Muscle. Br. J. Sports Med..

[3] Bailey S.J., Vanhatalo A., Winyard P.G., Jones A.M. (2012). The Nitrate-Nitrite-Nitric Oxide Pathway: Its Role in Human Exercise Physiology. Eur. J. Sport Sci..

[4] Besco R., Sureda A., Tur J.A., Pons A. (2012). The Effect of Nitric-Oxide-Related Supplements on Human Performance. Sports Med..

[5] Pérez-Guisado J., Jakeman P.M. (2010). Citrulline Malate Enhances Athletic Anaerobic Performance and Relieves Muscle Soreness. J. Strength Cond. Res..

[6] Rhim H.C., Kim S.J., Park J., Jang K.-M. (2020). Effect of Citrulline on Post-Exercise Rating of Perceived Exertion, Muscle Soreness, and Blood Lactate Levels: A Systematic Review and Meta-Analysis. J. Sport Health Sci..

[7] Bayat D., Azizi M., Behpour N., Tinsley G.M. (2025). Changes in Resistance Training Performance, Rating of Perceived Exertion, and Blood Biomarkers after Six Weeks of Supplementation with L-Citrulline vs. L-Citrulline DL-Malate in Resistance-Trained Men: A Double-Blind Placebo-Controlled Trial. J. Int. Soc. Sports Nutr..

[8] Chappell A.J., Parry A., Simper T. (2024). The Acute Effect of Citrulline Malate Loading in Resistance Trained Males on: Anaerobic Muscular Endurance, Force Recovery and Muscle Soreness. J. Sci. Sport Exerc..

[9] Sharma N., Shori G., Jaipuriar D.S. (2014). Effects of Single Dose of Citrulline Malate on Performance in Collegiate Male Athltes. Rom. J. Phys. Ther..

[10] Bezuglov E., Morgans R., Lazarev A., Kalinin E., Butovsky M., Savin E., Tzgoev E., Pirmakhanov B., Emanov A., Zholinsky A. (2022). The Effect of a Single Dose of Citrulline on the Physical Performance of Soccer-Specific Exercise in Adult Elite Soccer Players (A Pilot Randomized Double-Blind Trial). Nutrients.

[11] Chappell A.J., Allwood D.M., Johns R., Brown S., Sultana K., Anand A., Simper T. (2018). Citrulline Malate Supplementation Does Not Improve German Volume Training Performance or Reduce Muscle Soreness in Moderately Trained Males and Females. J. Int. Soc. Sports Nutr..

[12] Chappell A.J., Allwood D.M., Simper T.N. (2020). Citrulline Malate Fails to Improve German Volume Training Performance in Healthy Young Men and Women. J. Diet. Suppl..

[13] Harnden C.S., Agu J., Gascoyne T. (2023). Effects of Citrulline on Endurance Performance in Young Healthy Adults: A Systematic Review and Meta-Analysis. J. Int. Soc. Sports Nutr..

[14] Viribay A., Fernández-Landa J., Castañeda-Babarro A., Collado P.S., Fernández-Lázaro D., Mielgo-Ayuso J. (2022). Effects of Citrulline Supplementation on Different Aerobic Exercise Performance Outcomes: A Systematic Review and Meta-Analysis. Nutrients.

[15] Vårvik F.T., Bjørnsen T., Gonzalez A.M. (2021). Acute Effect of Citrulline Malate on Repetition Performance during Strength Training: A Systematic Review and Meta-Analysis. Int. J. Sport Nutr. Exerc. Metab..

[16] Aguiar A.F., Casonatto J. (2022). Effects of Citrulline Malate Supplementation on Muscle Strength in Resistance-Trained Adults: A Systematic Review and Meta-Analysis of Randomized Controlled Trials. J. Diet. Suppl..

[17] Gough L.A., Sparks S.A., McNaughton L.R., Higgins M.F., Newbury J.W., Trexler E., Faghy M.A., Bridge C.A. (2021). A Critical Review of Citrulline Malate Supplementation and Exercise Performance. Eur. J. Appl. Physiol..

[18] Nobari H., Samadian L., Saedmocheshi S., Prieto-González P., MacDonald C. (2025). Overview of Mechanisms Related to Citrulline Malate Supplementation and Different Methods of High-Intensity Interval Training on Sports Performance: A Narrative Review. Heliyon.

[19] Page M.J., McKenzie J.E., Bossuyt P.M., Boutron I., Hoffmann T.C., Mulrow C.D., Shamseer L., Tetzlaff J.M., Akl E.A., Brennan S.E. (2021). The PRISMA 2020 Statement: An Updated Guideline for Reporting Systematic Reviews. BMJ.

[20] Premaratne S., Newman J., Hobbs S., Garnham A., Wall M. (2020). Meta-Analysis of Direct Surgical versus Endovascular Revascularization for Aortoiliac Occlusive Disease. J. Vasc. Surg..

[21] Grgic J. (2018). Caffeine Ingestion Enhances Wingate Performance: A Meta-Analysis. Eur. J. Sport Sci..

[22] Sterne J.A.C., Savović J., Page M.J., Elbers R.G., Blencowe N.S., Boutron I., Cates C.J., Cheng H.-Y., Corbett M.S., Eldridge S.M. (2019). RoB 2: A Revised Tool for Assessing Risk of Bias in Randomised Trials. BMJ.

[23] Higgins J., Thomas J., Chandler J., Cumpston M., Li T., Page M.J., Welch V.A. (2019). Cochrane Handbook for Systematic Reviews of Interventions.

[24] Cumpston M., Li T., Page M.J., Chandler J., Welch V.A., Higgins J.P., Thomas J. (2019). Updated Guidance for Trusted Systematic Reviews: A New Edition of the Cochrane Handbook for Systematic Reviews of Interventions. Cochrane Database Syst. Rev..

[25] Hedges L.V., Shymansky J.A., Woodworth G. (1989). A Practical Guide to Modern Methods of Meta-Analysis.

[26] Hedges L.V. (1983). A Random Effects Model for Effect Sizes. Psychol. Bull..

[27] Cohen J. (2013). Statistical Power Analysis for the Behavioral Sciences.

[28] Cheung M.W.-L. (2019). A Guide to Conducting a Meta-Analysis with Non-Independent Effect Sizes. Neuropsychol. Rev..

[29] Assink M., Wibbelink C. (2016). Fitting Three-Level Meta-Analytic Models in R: A Step-by-Step Tutorial. Quant. Methods Psychol..

[30] Kadlec D., Sainani K.L., Nimphius S. (2023). With Great Power Comes Great Responsibility: Common Errors in Meta-Analyses and Meta-Regressions in Strength & Conditioning Research. Sports Med..

[31] Van den Noortgate W., López-López J.A., Marín-Martínez F., Sánchez-Meca J. (2013). Three-Level Meta-Analysis of Dependent Effect Sizes. Behav. Res..

[32] Jukic I., Castilla A.P., Ramos A.G., Van Hooren B., McGuigan M.R., Helms E.R. (2023). The Acute and Chronic Effects of Implementing Velocity Loss Thresholds during Resistance Training: A Systematic Review, Meta-Analysis, and Critical Evaluation of the Literature. Sports Med..

[33] Borg D.N., Impellizzeri F.M., Borg S.J., Hutchins K.P., Stewart I.B., Jones T., Baguley B.J., Orssatto L.B.R., Bach A.J.E., Osborne J.O. (2024). Meta-Analysis Prediction Intervals Are under Reported in Sport and Exercise Medicine. Scand. J. Med. Sci. Sports.

[34] IntHout J., Ioannidis J.P.A., Rovers M.M., Goeman J.J. (2016). Plea for Routinely Presenting Prediction Intervals in Meta-Analysis. BMJ Open.

[35] Nakagawa S., Noble D.W.A., Senior A.M., Lagisz M. (2017). Meta-Evaluation of Meta-Analysis: Ten Appraisal Questions for Biologists. BMC Biol..

[36] Quintana D.S. (2023). A Guide for Calculating Study-Level Statistical Power for Meta-Analyses. Adv. Methods Pract. Psychol. Sci..

[37] Hopkins W.G. (2018). Improving Meta-Analyses in Sport and Exercise Science. Sportscience.

[38] Deeks J.J., Higgins J.P., Altman D.G. (2019). on behalf of the Cochrane Statistical Methods Group. Analysing Data and Undertaking Meta-Analyses. Cochrane Handbook for Systematic Reviews of Interventions.

[39] Ruppar T. (2020). Meta-Analysis: How to Quantify and Explain Heterogeneity?. Eur. J. Cardiovasc. Nurs..

[40] McKay A.K.A., Stellingwerff T., Smith E.S., Martin D.T., Mujika I., Goosey-Tolfrey V.L., Sheppard J., Burke L.M. (2022). Defining Training and Performance Caliber: A Participant Classification Framework. Int. J. Sports Physiol. Perform..

[41] Deng H., Song T., Yin M., Xu K., Zhong Y., Liu P., Sun S., Bin Naharudin M.N., Yusof A., Fan X. (2025). Does One Shot Work? The Acute Impact of a Single Taurine Dose on Exercise Performance: A Meta-Analytic Review. Scand. J. Med. Sci. Sports.

[42] Riebe D., Ehrman J.K., Liguori G., Magal M. (2018). ACSM’s Guidelines for Exercise Testing and Prescription.

[43] Zuur A.F., Ieno E.N., Walker N., Saveliev A.A., Smith G.M. (2009). Mixed Effects Models and Extensions in Ecology with R; Statistics for Biology and Health.

[44] Harrell F.E., Harrell F.E. (2015). General Aspects of Fitting Regression Models. Regression Modeling Strategies: With Applications to Linear Models, Logistic and Ordinal Regression, and Survival Analysis.

[45] Wickham H. (2011). Ggplot2. WIREs Comput. Stat..

[46] Peters J.L., Sutton A.J., Jones D.R., Abrams K.R., Rushton L. (2008). Contour-Enhanced Meta-Analysis Funnel Plots Help Distinguish Publication Bias from Other Causes of Asymmetry. J. Clin. Epidemiol..

[47] Egger M., Smith G.D., Schneider M., Minder C. (1997). Bias in Meta-Analysis Detected by a Simple, Graphical Test. BMJ.

[48] Fernández-Castilla B., Declercq L., Jamshidi L., Beretvas S.N., Onghena P., Noortgate W.V.D. (2021). Detecting Selection Bias in Meta-Analyses with Multiple Outcomes: A Simulation Study. J. Exp. Educ..

[49] Sterne J.A.C., Sutton A.J., Ioannidis J.P.A., Terrin N., Jones D.R., Lau J., Carpenter J., Rücker G., Harbord R.M., Schmid C.H. (2011). Recommendations for Examining and Interpreting Funnel Plot Asymmetry in Meta-Analyses of Randomised Controlled Trials. BMJ.

[50] Viechtbauer W., Cheung M.W.-L. (2010). Outlier and Influence Diagnostics for Meta-Analysis. Res. Synth. Methods.

[51] Atkinson A.C., Cook R.D., Weisberg S. (1983). Residuals and Influence in Regression. Biometrics.

[52] Schünemann H.J., Higgins J.P., Vist G.E., Glasziou P., Akl E.A., Skoetz N., Guyatt G.H. (2019). Completing ‘Summary of Findings’ Tables and Grading the Certainty of the Evidence. Cochrane Handbook for Systematic Reviews of Interventions.

[53] Cunniffe B., Papageorgiou M., O’Brien B., Davies N.A., Grimble G.K., Cardinale M. (2016). Acute Citrulline-Malate Supplementation and High-Intensity Cycling Performance. J. Strength Cond. Res..

[54] Devrim-Lanpir A., Ihász F., Demcsik M., Horváth A.C., Góczán P., Czepek P., Takács J., Kimble R., Zare R., Gunes F.E. (2024). Effects of Acute Citrulline Malate Supplementation on CrossFit^®^ Exercise Performance: A Randomized, Double-Blind, Placebo-Controlled, Cross-over Study. Nutrients.

[55] Faria V.S., Egan B. (2024). Effects of 3 Days of Citrulline Malate Supplementation on Short-Duration Repeated Sprint Running Performance in Male Team Sport Athletes. Eur. J. Sport Sci..

[56] Farney T.M., Bliss M.V., Hearon C.M., Salazar D.A. (2019). The Effect of Citrulline Malate Supplementation on Muscle Fatigue among Healthy Participants. J. Strength Cond. Res..

[57] Fick A.N., Kowalsky R.J., Stone M.S., Hearon C.M., Farney T.M. (2021). Acute and Chronic Citrulline Malate Supplementation on Muscle Contractile Properties and Fatigue Rate of the Quadriceps. Int. J. Sport Nutr. Exerc. Metab..

[58] Gills J.L., Glenn J.M., Gray M., Romer B., Lu H. (2021). Acute Citrulline-Malate Supplementation Is Ineffective during Aerobic Cycling and Subsequent Anaerobic Performance in Recreationally Active Males. Eur. J. Sport Sci..

[59] Gills J.L., Spliker B., Glenn J.M., Szymanski D., Romer B., Lu H.-C., Gray M. (2023). Acute Citrulline-Malate Supplementation Increases Total Work in Short Lower-Body Isokinetic Tasks for Recreationally Active Females during Menstruation. J. Strength Cond. Res..

[60] Glenn J.M., Gray M., Jensen A., Stone M.S., Vincenzo J.L. (2016). Acute Citrulline-Malate Supplementation Improves Maximal Strength and Anaerobic Power in Female, Masters Athletes Tennis Players. Eur. J. Sport Sci..

[61] Glenn J.M., Gray M., Wethington L.N., Stone M.S., Stewart R.W., Moyen N.E. (2017). Acute Citrulline Malate Supplementation Improves Upper- and Lower-Body Submaximal Weightlifting Exercise Performance in Resistance-Trained Females. Eur. J. Nutr..

[62] Gonzalez A.M., Spitz R.W., Ghigiarelli J.J., Sell K.M., Mangine G.T. (2018). Acute Effect of Citrulline Malate Supplementation on Upper-Body Resistance Exercise Performance in Recreationally Resistance-Trained Men. J. Strength Cond. Res..

[63] Grala A., Candellório É., Sperandio P., Maldonado E., Anjos B.D., Jacinto J., Casonatto J., Aguiar A. (2021). Effects of citrulline malate supplementation on aerobic and muscular endurance in young adults men. J. Health Sci..

[64] Haugen M.E., Vårvik F.T., Grgic J., Studsrud H., Austheim E., Zimmermann E.M., Falch H.N., Larsen S., van den Tillaar R., Bjørnsen T. (2023). Effect of Isolated and Combined Ingestion of Caffeine and Citrulline Malate on Resistance Exercise and Jumping Performance: A Randomized Double-Blind Placebo-Controlled Crossover Study. Eur. J. Nutr..

[65] Hwang P., Morales Marroquín F.E., Gann J., Andre T., McKinley-Barnard S., Kim C., Morita M., Willoughby D.S. (2018). Eight Weeks of Resistance Training in Conjunction with Glutathione and L-Citrulline Supplementation Increases Lean Mass and Has No Adverse Effects on Blood Clinical Safety Markers in Resistance-Trained Males. J. Int. Soc. Sports Nutr..

[66] Jafari R.A., Hosseini S.R.A., Rashidlamir A., Nobari H. (2024). Evaluating the Impact of Active and Passive Recovery Strategies and Citrulline-Malate Supplementation in Wrestling: Do the Results Add Up?. Acta Kinesiol..

[67] Martín-Olmedo J.J., Miras-Moreno S., Cuadra-Montes K., García-Ramos A., Ruiz J.R., Jurado-Fasoli L. (2024). Malate or Not? Acute Effects of L-Citrulline versus Citrulline Malate on Neuromuscular Performance in Young, Trained Adults: A Randomized, Double-Blind, Placebo-Controlled Crossover Trial. Int. J. Sport Nutr. Exerc. Metab..

[68] Mayo J., Lyons B.C., Tucker W.S., Wax B. (2023). Acute Citrulline Malate Supplementation Does Not Improve Anaerobic Capacity in Healthy Young Adults: A Pilot Study. J. Exerc. Nutr..

[69] Naimah N.M., Linoby A., Norhamazi I., Haslan A.N., Zubir S.M.S., Noor M.A.M., Zamri F.N.S. (2022). Influence of Acute Ctrulline-Malate Supplementation on Maximal Strength and Anaerobic Power in Combat Athletes. J. Phys. Educ. Sport.

[70] Newbury J.W., Cole M., Bailey S.J., Kelly A.L., Gough L.A. (2024). Citrulline Malate Fails to Improve Repeated 300 m Swimming Times in Highly Trained Swimmers. Physiologia.

[71] Tishchenko A.A., Kaplanyan D.A., Krechetova V.A., Frantsuzov Y.K., Shakhbanov I.S., Chnavayan A.V. (2023). Effectiveness of the Use of Citrulline Malate to Increase the Powerlifters Adaptive Potential and Physical Performance. J. Biochem. Technol..

[72] Viana J.C.B., Azevedo A.P., Freire de Almeida R., Vicentini G., Barauna V.G., Guimaraes-Ferreira L. (2021). Acute Citrulline-Malate Ingestion Does Not Enhance Performance in Judo Athletes. Ido Mov. Cult. J. Martial Arts Anthropol..

[73] Wax B., Kavazis A.N., Weldon K., Sperlak J. (2015). Effects of Supplemental Citrulline Malate Ingestion during Repeated Bouts of Lower-Body Exercise in Advanced Weightlifters. J. Strength Cond. Res..

[74] Wax B., Kavazis A.N., Luckett W. (2016). Effects of Supplemental Citrulline-Malate Ingestion on Blood Lactate, Cardiovascular Dynamics, and Resistance Exercise Performance in Trained Males. J. Diet. Suppl..

[75] Morris S.M., Ignarro L.J. (2000). Chapter 11—Regulation of Arginine Availability and Its Impact on NO Synthesis. Nitric Oxide.

[76] Böger R.H. (2004). Asymmetric Dimethylarginine, an Endogenous Inhibitor of Nitric Oxide Synthase, Explains the “L-Arginine Paradox” and Acts as a Novel Cardiovascular Risk Factor. J. Nutr..

[77] Förstermann U., Sessa W.C. (2012). Nitric Oxide Synthases: Regulation and Function. Eur. Heart J..

[78] Bailey S.J., Blackwell J.R., Lord T., Vanhatalo A., Winyard P.G., Jones A.M. (2015). L-Citrulline Supplementation Improves O_2_ Uptake Kinetics and High-Intensity Exercise Performance in Humans. J. Appl. Physiol..

[79] Suzuki T., Morita M., Kobayashi Y., Kamimura A. (2016). Oral L-Citrulline Supplementation Enhances Cycling Time Trial Performance in Healthy Trained Men: Double-Blind Randomized Placebo-Controlled 2-Way Crossover Study. J. Int. Soc. Sports Nutr..

[80] Moreau K.L., Hildreth K.L., Meditz A.L., Deane K.D., Kohrt W.M. (2012). Endothelial Function Is Impaired across the Stages of the Menopause Transition in Healthy Women. J. Clin. Endocrinol. Metab..

[81] Stanhewicz A.E., Wenner M.M., Stachenfeld N.S. (2018). Sex Differences in Endothelial Function Important to Vascular Health and Overall Cardiovascular Disease Risk across the Lifespan. Am. J. Physiol.-Heart Circ. Physiol..

[82] Williams M.R.I., Westerman R.A., Kingwell B.A., Paige J., Blombery P.A., Sudhir K., Komesaroff P.A. (2001). Variations in Endothelial Function and Arterial Compliance during the Menstrual Cycle. J. Clin. Endocrinol. Metab..

[83] Hunter S.K. (2014). Sex Differences in Human Fatigability: Mechanisms and Insight to Physiological Responses. Acta Physiol..

[84] Green D.J., Spence A., Rowley N., Thijssen D.H.J., Naylor L.H. (2012). Vascular Adaptation in Athletes: Is There an ‘Athlete’s Artery’?. Exp. Physiol..

[85] Laughlin M.H., Roseguini B. (2008). Mechanisms for Exercise Training-Induced Increases in Skeletal Muscle Blood Flow Capacity: Differences with Interval Sprint Training versus Aerobic Endurance Training. J. Physiol. Pharmacol..

[86] Ribas M.R., Schneider F.K., Ribas D.I.R., Badicu G., Bonatto A.C., Ardigò L.P., Bassan J.C. (2024). Impact of Genetic Polymorphisms on Electrochemical Parameters and Acid-Base Disorders in Brazilian Runners during a 105-Kilometer Ultramarathon. Nutrients.

[87] Gonzalez A.M., Trexler E.T. (2020). Effects of Citrulline Supplementation on Exercise Performance in Humans: A Review of the Current Literature. J. Strength Cond. Res..

[88] Moinard C., Nicolis I., Neveux N., Darquy S., Bénazeth S., Cynober L. (2008). Dose-Ranging Effects of Citrulline Administration on Plasma Amino Acids and Hormonal Patterns in Healthy Subjects: The Citrudose Pharmacokinetic Study. Br. J. Nutr..

[89] Davis A.R., Webber C.L., Fish W.W., Wehner T.C., King S., Perkins-Veazie P. (2011). L-Citrulline Levels in Watermelon Cultigens Tested in Two Environments. HortScience.

