# Supplementary material for: Effects of Citrulline Malate Supplementation on Exercise Performance: A Systematic Review and Three-Level Meta-Analysis
Source: Nutrients. 2026 Jun 11;18(12):1881. doi: 10.3390/nu18121881 (PMC13304508; doi:10.3390/nu18121881)
Supplement: Supplementary file 1 [file nutrients-18-01881-s001.zip › nutrients-4346197-supplementary.pdf]

Electronic Supplementary Material Contents

Number	Material	Page
1	Electronic Supplementary Material S1 (PRISMA 2020 Checklist)	P2-5
2	Electronic Supplementary Material S2 (Search Records)	6
3	Electronic Supplementary Material S3 (Table. Summary of Effect Size Calculation Procedures)	P7-8
4	Electronic Supplementary Material S4 (GRADE Assessment)	P9-13
5	Electronic Supplementary Material S5 (Summary Forest Plot of Aggregated Study effects)	P14-16
6	Electronic Supplementary Material S6 (Supplementary Table: Numerical results of subgroup and moderator analyses)	P17-18
7	Electronic Supplementary Material S7 ((ROB2 Assessment Tool for Risk of Bias)	P19
8	Electronic Supplementary Material S8 (Funnel plot)	P20-21
9	Electronic Supplementary Material S9 (Power Visualization)	P22-23
10	Electronic Supplementary Material S10 ((PEDro Assessment)	P24-25
11	Electronic Supplementary Material S11 (Summary of Sensitivity Analysis Results)	P26-27
12	Electronic Supplementary Material S12 (A Sensitivity Analysis Based on Level-3 Leave-one-out)	P28-30
13	Electronic Supplementary Material S13 (Moderator Analysis After Excluding Outliers)	P31
14	Electronic Supplementary Material S14 (Regression Analysis After Excluding Outliers)	P32

Electronic Supplementary Material S1 (PRISMA 2020 Checklist)

Section and Topic	Item #	Checklist item	Location where item is reported (on page)
**TITLE: Effects of Citrulline Malate Supplementation on Exercise Performance: A Systematic Review and Three-Level Meta-Analysis**			
Title	1	Identify the report as a systematic review.	P1
**ABSTRACT**			
Abstract	2	See the PRISMA 2020 for Abstracts checklist.	P1-2
**INTRODUCTION**			
Rationale	3	Describe the rationale for the review in the context of existing knowledge.	P2-3
Objectives	4	Provide an explicit statement of the objective(s) or question(s) the review addresses.	P2-3
**METHODS**			
Eligibility criteria	5	Specify the inclusion and exclusion criteria for the review and how studies were grouped for the syntheses.	P3
Information sources	6	Specify all databases, registers, websites, organisations, reference lists and other sources searched or consulted to identify studies. Specify the date when each source was last searched or consulted.	P3-4
Search strategy	7	Present the full search strategies for all databases, registers and websites, including any filters and limits used.	P3-4
Selection process	8	Specify the methods used to decide whether a study met the inclusion criteria of the review, including how many reviewers screened each record and each report retrieved, whether they worked independently, and if applicable, details of automation tools used in the process.	P3-4
Data collection process	9	Specify the methods used to collect data from reports, including how many reviewers collected data from each report, whether they worked independently, any processes for obtaining or confirming data from study investigators, and if applicable, details of automation tools used in the process.	P3-4
Data items	10a	List and define all outcomes for which data were sought. Specify whether all results that were compatible with each outcome domain in each study were sought (e.g. for all measures, time points, analyses), and if not, the methods used to decide which results to collect.	P4
	10b	List and define all other variables for which data were sought (e.g. participant and intervention characteristics, funding sources). Describe any assumptions made about any missing or unclear information.	P4

Section and Topic	Item #	Checklist item	Location where item is reported (on page)
Study risk of bias assessment	11	Specify the methods used to assess risk of bias in the included studies, including details of the tool(s) used, how many reviewers assessed each study and whether they worked independently, and if applicable, details of automation tools used in the process.	P4
Effect measures	12	Specify for each outcome the effect measure(s) (e.g. risk ratio, mean difference) used in the synthesis or presentation of results.	P4-5
Synthesis methods	13a	Describe the processes used to decide which studies were eligible for each synthesis (e.g. tabulating the study intervention characteristics and comparing against the planned groups for each synthesis (item #5)).	P5
	13b	Describe any methods required to prepare the data for presentation or synthesis, such as handling of missing summary statistics, or data conversions.	P5
	13c	Describe any methods used to tabulate or visually display results of individual studies and syntheses.	P4-6
	13d	Describe any methods used to synthesize results and provide a rationale for the choice(s). If meta-analysis was performed, describe the model(s), method(s) to identify the presence and extent of statistical heterogeneity, and software package(s) used.	P5-6
	13e	Describe any methods used to explore possible causes of heterogeneity among study results (e.g. subgroup analysis, meta-regression).	P6-7
	13f	Describe any sensitivity analyses conducted to assess robustness of the synthesized results.	P6-7
Reporting bias assessment	14	Describe any methods used to assess risk of bias due to missing results in a synthesis (arising from reporting biases).	P6-7
Certainty assessment	15	Describe any methods used to assess certainty (or confidence) in the body of evidence for an outcome.	P7
**RESULTS**			
Study selection	16a	Describe the results of the search and selection process, from the number of records identified in the search to the number of studies included in the review, ideally using a flow diagram.	P7-8
	16b	Cite studies that might appear to meet the inclusion criteria, but which were excluded, and explain why they were excluded.	P7-8
Study characteristics	17	Cite each included study and present its characteristics.	P8-13
Risk of bias in	18	Present assessments of risk of bias for each included study.	P17-18

Section and Topic	Item #	Checklist item	Location where item is reported (on page)
studies			
Results of individual studies	19	For all outcomes, present, for each study: (a) summary statistics for each group (where appropriate) and (b) an effect estimate and its precision (e.g. confidence/credible interval), ideally using structured tables or plots.	P17-19
Results of syntheses	20a	For each synthesis, briefly summarise the characteristics and risk of bias among contributing studies.	P20
	20b	Present results of all statistical syntheses conducted. If meta-analysis was done, present for each the summary estimate and its precision (e.g. confidence/credible interval) and measures of statistical heterogeneity. If comparing groups, describe the direction of the effect.	P18-19
	20c	Present results of all investigations of possible causes of heterogeneity among study results.	P13-17
	20d	Present results of all sensitivity analyses conducted to assess the robustness of the synthesized results.	P13-17
Reporting biases	21	Present assessments of risk of bias due to missing results (arising from reporting biases) for each synthesis assessed.	P17-18
Certainty of evidence	22	Present assessments of certainty (or confidence) in the body of evidence for each outcome assessed.	P17-18
**DISCUSSION**			
Discussion	23a	Provide a general interpretation of the results in the context of other evidence.	P18-21
	23b	Discuss any limitations of the evidence included in the review.	P22-23
	23c	Discuss any limitations of the review processes used.	P22-23
	23d	Discuss implications of the results for practice, policy, and future research.	P21-22
**OTHER INFORMATION**			
Registration and protocol	24a	Provide registration information for the review, including register name and registration number, or state that the review was not registered.	P3
	24b	Indicate where the review protocol can be accessed, or state that a protocol was not prepared.	P3
	24c	Describe and explain any amendments to information provided at registration or in the protocol.	N/A
Support	25	Describe sources of financial or non-financial support for the review, and the role of the funders or sponsors in the review.	P23-24
Competing	26	Declare any competing interests of review authors.	P23-24

Section and Topic	Item #	Checklist item	Location where item is reported (on page)
interests			
Availability of data, code and other materials	27	Report which of the following are publicly available and where they can be found: template data collection forms; data extracted from included studies; data used for all analyses; analytic code; any other materials used in the review.	P3

Electronic Supplementary Material S2 (Search Records)

Data	Query	Limits	Results
**PUBMED**	("citrulline malate"[Title/Abstract] OR "citrulline-malate"[Title/Abstract] OR "L-citrulline malate"[Title/Abstract] OR "L-citrulline DL-malate"[Title/Abstract] OR (citrulline[Title/Abstract] AND malate[Title/Abstract])) AND ("exercise performance"[Title/Abstract] OR endurance[Title/Abstract] OR "aerobic capacity"[Title/Abstract] OR "anaerobic capacity"[Title/Abstract] OR strength[Title/Abstract] OR "resistance training"[Title/Abstract] OR VO2max[Title/Abstract] OR fatigue[Title/Abstract] OR "time to exhaustion"[Title/Abstract])	No	52
**Web of Science**	("citrulline malate" OR "citrulline-malate" OR "L-citrulline malate" OR "L-citrulline DL-malate" OR (citrulline AND malate)) AND ("exercise performance" OR endurance OR "aerobic capacity" OR "anaerobic capacity" OR strength OR "resistance training" OR VO2max OR fatigue OR "time to exhaustion")	No	84
**Cochrane Library**	("citrulline malate":ti,ab,kw OR "citrulline-malate":ti,ab,kw OR "L-citrulline malate":ti,ab,kw OR "L-citrulline DL-malate":ti,ab,kw OR (citrulline:ti,ab,kw AND malate:ti,ab,kw)) AND ("exercise performance":ti,ab,kw OR endurance:ti,ab,kw OR "aerobic capacity":ti,ab,kw OR "anaerobic capacity":ti,ab,kw OR strength:ti,ab,kw OR "resistance training":ti,ab,kw OR VO2max:ti,ab,kw OR fatigue:ti,ab,kw OR "time to exhaustion":ti,ab,kw)	No	42
**Embase**	(('citrulline malate':ti,ab,kw OR 'citrulline-malate':ti,ab,kw OR 'l-citrulline malate':ti,ab,kw OR 'l-citrulline dl-malate':ti,ab,kw OR (citrulline:ti,ab,kw AND malate:ti,ab,kw)) AND ('exercise performance':ti,ab,kw OR endurance:ti,ab,kw OR 'aerobic capacity':ti,ab,kw OR 'anaerobic capacity':ti,ab,kw OR strength:ti,ab,kw OR 'resistance training':ti,ab,kw OR vo2max:ti,ab,kw OR fatigue:ti,ab,kw OR 'time to exhaustion':ti,ab,kw)	No	55
**SciELO**	("citrulline malate" OR "citrulline-malate" OR "L-citrulline malate" OR "L-citrulline DL-malate" OR (citrulline AND malate)) AND ("exercise performance" OR endurance OR "aerobic capacity" OR "anaerobic capacity" OR strength OR "resistance training" OR VO2max OR fatigue OR "time to exhaustion")	No	0
**SPORTDiscus**	("citrulline malate" OR "citrulline-malate" OR "L-citrulline malate" OR "L-citrulline DL-malate" OR (citrulline AND malate)) AND TX ("exercise performance" OR endurance OR "aerobic capacity" OR "anaerobic capacity" OR strength OR "resistance training" OR VO2max OR fatigue OR "time to exhaustion")	No	34

Electronic Supplementary Material S3 (Table. Summary of Effect Size Calculation Procedures)

Step	Data condition	Calculation Formula	Description
①	Pre–post data available	$\Delta\mathrm{MEN} = \mathrm{M}_{\mathrm{CMpost}} - \mathrm{M}_{\mathrm{CMpre}}$<br>$\Delta\mathrm{PLA} = \mathrm{M}_{\mathrm{PLApost}} - \mathrm{M}_{\mathrm{PLApre}}$<br>$\mathrm{MD} = \Delta\mathrm{CM} - \Delta\mathrm{PLA}$	Calculate the mean change within each condition (CM and placebo) and use their difference (MD) as the comparison metric.
②	Only post-intervention data available	$\mathrm{MD} = \mathrm{M}_{\mathrm{CM}} - \mathrm{M}_{\mathrm{PLA}}$	When only post values are reported, the mean difference between conditions is used directly.
③	SD of change (for pre–post data)	$\mathrm{SD}_{\mathrm{change}} = \sqrt{\mathrm{SD}^2{}_{\mathrm{pre}} + \mathrm{SD}^2{}_{\mathrm{post}} - \left(2 \times r \times \mathrm{SD}_{\mathrm{pre}} \times \mathrm{SD}_{\mathrm{post}}\right)}$	Computes the within-subject SD of change using the correlation (r) between pre- and post-measurements.
④	Pooled SD for (*i*) crossover and (*ii*) parallel design	$\mathrm{SD}_{\mathrm{pooled}} = \sqrt{\frac{\mathrm{SD}^2{}_{\mathrm{CM}} + \mathrm{SD}^2{}_{\mathrm{PLA}}}{2}}$ *(i)*<br>$\mathrm{SD}_{\mathrm{pooled}} = \sqrt{\frac{(n_{\mathrm{CM}}-1) \times SD^2_{\mathrm{CM}} + (n_{\mathrm{PLA}}-1) \times SD^2_{\mathrm{PLA}}}{n_{\mathrm{CM}} + n_{\mathrm{PLA}} - 2}}$ (*ii*)	Estimates the pooled variability across both conditions.
⑤		$\text{Hedges' g} = \frac{\mathrm{M}_{\mathrm{CM}} - \mathrm{M}_{\mathrm{PLA}}}{\mathrm{SD}_{\mathrm{pooled}}} \times \left(1 - \frac{3}{4(N-1)-1}\right)$ *(i)*	Standardized mean difference corrected for small-sample bias.

	Effect size (Hedges' *g*) for (*i*) crossover and (*ii*) parallel design	$\text{Hedges' g} = \frac{\text{M}_{\text{CM}} - \text{M}_{\text{PLA}}}{\text{SD}_{\text{pooled}}} \times \left(1 - \frac{3}{4(\text{n}_{\text{CM}} + \text{n}_{\text{PLA}} - 2) - 1}\right)$ (*ii*)	
⑥	Standard error (SE) of *g* for (*i*) crossover and (*ii*) parallel design	$\text{SE} = \sqrt{\frac{1}{\text{N}} + \frac{\text{g}^2}{2\text{N}}} \times \sqrt{2(1-r)}$ (i)<br>$\text{SE} = \sqrt{\frac{n_{CM} + n_{PLA}}{n_{CM} \times n_{PLA}} + \frac{g^2}{2(n_{CM} + n_{PLA})}}$ (*ii*)	(*i*) Adjusts SE to account for within-subject correlation between CM and placebo conditions. (*ii*) Calculates SE from independent CM and placebo group sizes, with the harmonic mean accounting for potential imbalance.
⑦	Assumed correlation (*r*)	$r = 0.50$ (primary analysis)<br>$r = 0.20$ and 0.80 (sensitivity)	The same r value was used for both pre－post and between-condition comparisons to ensure consistency and comparability.

Electronic Supplementary Material S4 (GRADE Assessment)

Outcome	K	Certainty of Evidence Assessment					Hedges' g [95% CI] *	GRADE†
		Risk of Bias	Inconsistency	Indirectness	Imprecision	Others		
**Primary Outcome**								
Overall exercise performance	K = 138	Serious	Not serious	Not serious	Not serious	Publication bias	0.16 [0.05, 0.27] *	⊕⊕◯◯ Low
Overall (Acute) exercise performance	K = 114	Not serious	Serious	Not serious	Not serious	Publication bias	0.16 [0.04, 0.28] *	⊕⊕◯◯ Low
Overall (chronic) exercise performance	K = 24	Serious	Not serious	Not serious	Serious	Publication bias	0.15 [-0.06, 0.37]	⊕◯◯◯ Very low
Rating of perceived exertion	K = 17	Serious	Not serious	Not serious	Serious	None	-0.25 [-0.80, 0.29]	⊕⊕◯◯ Low
**Sex**								
Male	K = 69	Not serious	Serious	Not serious	Not serious	Publication bias	0.23 [0.04, 0.43] *	⊕⊕◯◯ Low
Female	K = 16	Very serious	Not serious	Not serious	Very serious		0.26 [-0.13, 0.65]	⊕◯◯◯

						None		Very low
Mixed	K = 29	Not serious	Not serious	Serious	Serious	None	0.01 [-0.25, 0.26]	⊕⊕◯◯ Low
**Training Level**								
Trained	K = 90	Not serious	Serious	Not serious	Not serious	Publication bias	0.22 [0.05, 0.38] *	⊕⊕◯◯ Low
Untrained	K = 24	Serious	Not serious	Not serious	Serious	None	0.03 [-0.23, 0.30]	⊕⊕◯◯ Low
**Exercise Type**								
Aerobic endurance	K = 14	Serious	Very serious	Not serious	Not serious	None	0.32 [0.02, 0.63] *	⊕◯◯◯ Very low
Anaerobic performance	K = 18	Not serious	Very serious	Not serious	Serious	None	0.28 [0.01, 0.55] *	⊕◯◯◯ Very low
Muscular endurance	K = 45	Not serious	Serious	Not serious	Serious	None	0.15 [-0.02, 0.32]	⊕⊕◯◯ Low
Strength/ Power	K = 37	Serious	Not serious	Not serious	Serious	None	0.10 [-0.08, 0.27]	⊕⊕◯◯

								Low
**Supplementation Timing**								
Before 40-45 minutes	K = 21	Not serious	Not serious	Serious	Very serious	None	-0.04 [-0.40, 0.32]	⊕◯◯◯ Very low
Before 60 minutes	K = 90	Serious	Not serious	Not serious	Not serious	Publication bias	0.22 [0.05, 0.38] *	⊕⊕◯◯ Low
Before 120 minutes	K = 3	Not serious	Not serious	Not serious	Extremely serious	None	-0.04 [-0.71, 0.63]	⊕◯◯◯ Very low
**Citrulline-Malate Dosage**								
4-4.4 g	K = 2	Not serious	Not serious	Not serious	Very serious	None	-0.06 [-0.75, 0.63]	⊕⊕◯◯ Low
8 g	K = 86	Not serious	Serious	Not serious	Not serious	Publication bias	0.20 [0.02, 0.38] *	⊕⊕◯◯ Low
12 g	K = 20	Not serious	Not serious	Not serious	Serious	Publication bias	0.08 [-0.44, 0.59]	⊕⊕◯◯ Low
15 g	K = 2	Serious	Not serious	Not serious	Extremely serious	None	0.08 [-0.76, 0.92]	⊕◯◯◯

								Very low
**Citrulline-Malate Form**								
Pill	K = 1	Not serious	Not serious	Not serious	Extremely serious	None	-0.05 [-0.82, 0.73]	⊕◯◯◯ Very low
Beverage	K = 113	Serious	Not serious	Not serious	Not serious	Publication bias	0.17 [0.02, 0.32] *	⊕⊕◯◯ Low

Notes: ***K*:** the total number of effects included in the pooled effect size; ***Publication bias***, represented by Egger test $p < 0.05$.

***:** The effect size (*Hedges'g*) was significant ($p < 0.05$).

† **GRADE Criteria for Certainty of Evidence**:
High: Very confident in the estimated effect.
Moderate: Moderately confident in the estimated effect.
Low: Limited confidence in the estimated effect.
Very low: Very limited confidence in the estimated effect.

Downgrading criteria: **(1)** Risk of bias was downgraded when a substantial proportion of contributing studies had risk-of-bias concerns. **(2)** Inconsistency was downgraded when heterogeneity was substantial or when prediction intervals suggested important variability in true effects. **(3)** Indirectness was downgraded when the population, intervention, comparator, or outcome had limited applicability to the main review question. **(4)** Imprecision was downgraded when confidence intervals were wide, crossed the null or clinically relevant thresholds, or when evidence was sparse. **(5)** Publication bias was downgraded when funnel-plot inspection or Egger's test suggested small-study effects or publication bias, where assessment was possible. For subgroup analyses, sparse evidence was considered primarily within the imprecision domain, while other GRADE domains were judged where applicable. Certainty ratings for sparse subgroups were reported for transparency and should be interpreted cautiously and mainly for descriptive purposes, rather than as precise or definitive certainty estimates for subgroup-specific effects or differences between subgroups.

Electronic Supplementary Material S5 (Summary Forest Plot of Aggregated Study Effects)

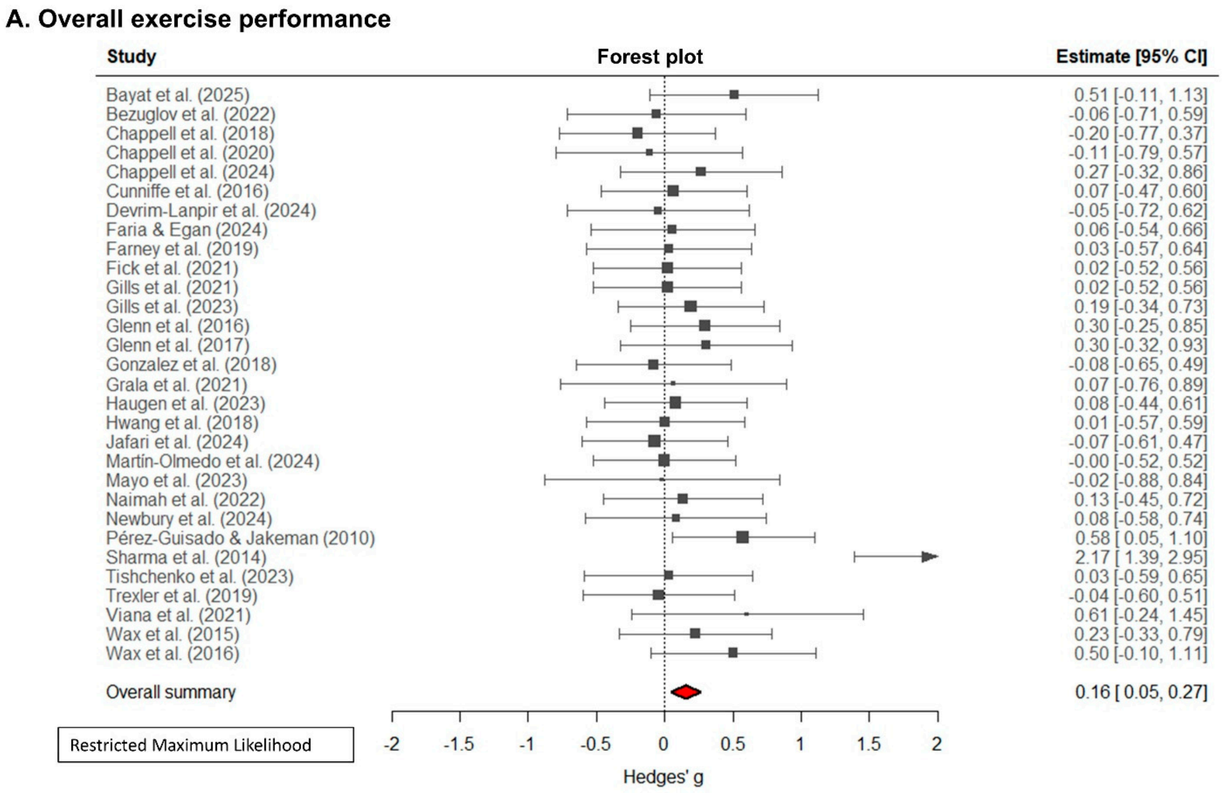

B. Exercise Performance_Chronic Intake

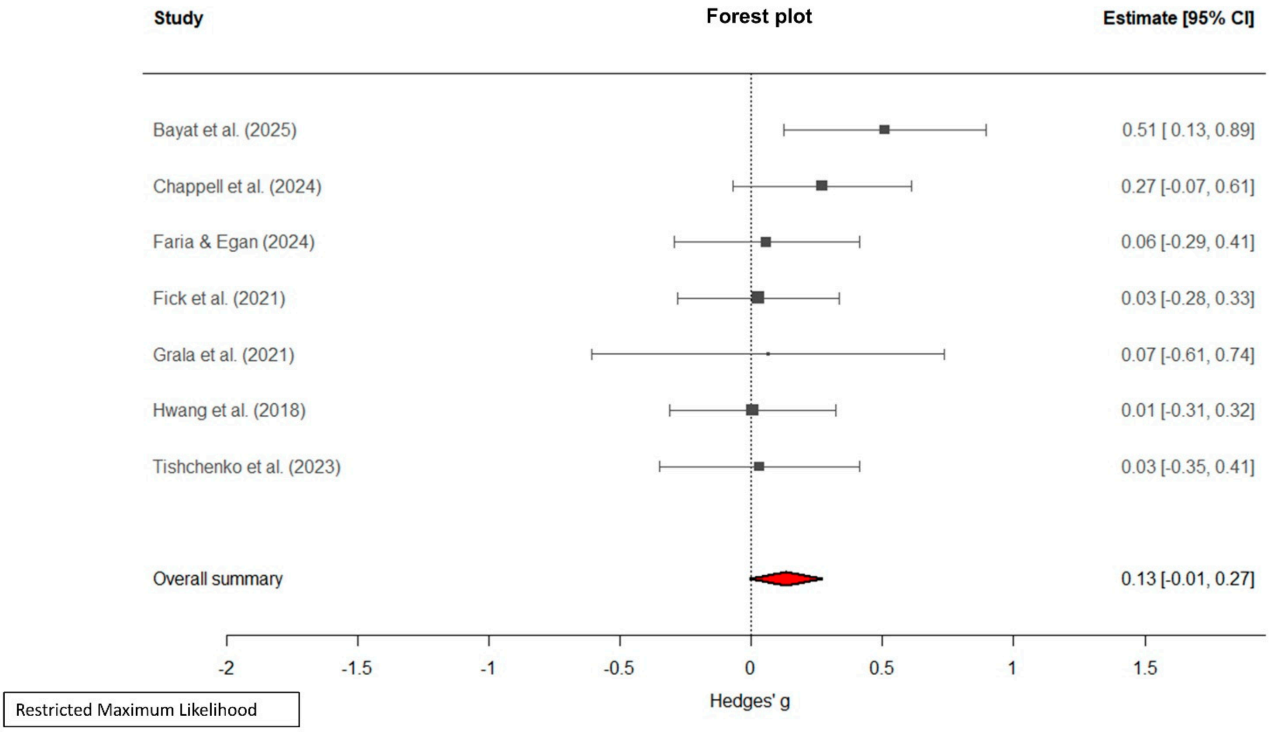

C. Exercise Performance_Acute Intake

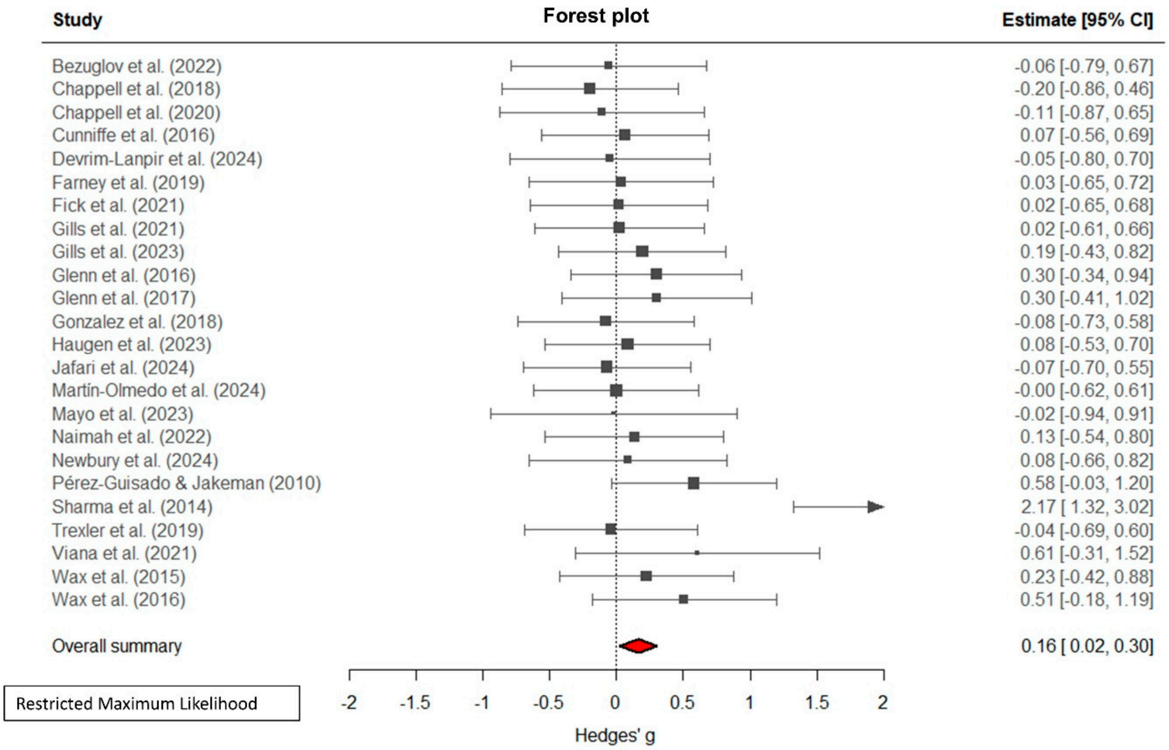

D. Rating of Perceived Exertion

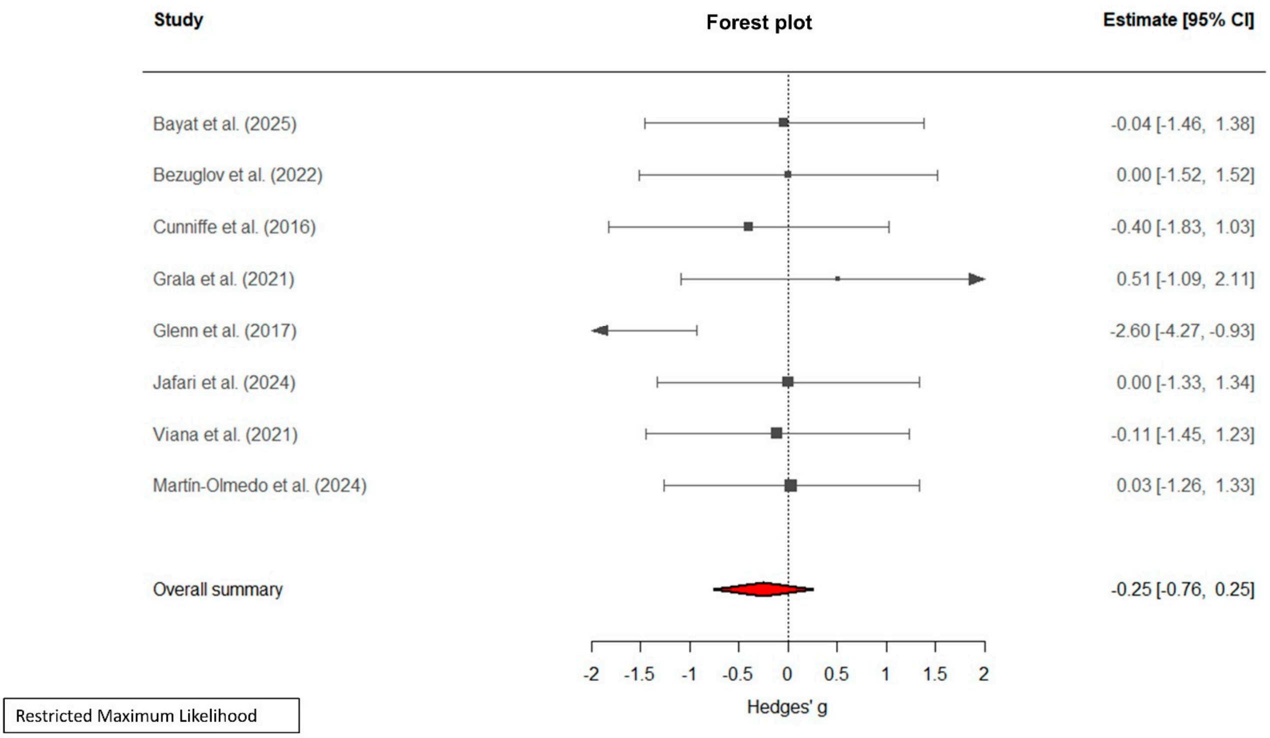

Notes: Hedges' g, the effect size indicators used in the pooled; ***95%CI***, 95% confidence interval; ***The size of each black square***, represents the relative weight of the study in the meta-analysis.

Electronic Supplementary Material S6 (Supplementary Table: Numerical Results of Subgroup and Moderator Analyses)

Moderator	Subgroup	k	Hedges' g	95% CI	PI	p value	I²	p_between	GRADE
Sex	Male	69	0.23	0.04 to 0.43	-0.43 to 0.90	0.02	65%	0.32	Low
Sex	Female	16	0.26	-0.13 to 0.65	-0.48 to 1.01	0.18	0	0.32	Very low
Sex	Mixed	29	0.01	-0.25 to 0.26	-0.68 to 0.69	0.97	0	0.32	Low
Training status	Trained	90	0.22	0.05 to 0.38	-0.44 to 0.87	0.01	60%	0.25	Low
Training status	Untrained	24	0.03	-0.23 to 0.30	-0.65 to 0.71	0.82	0	0.25	Low
Exercise category	Aerobic endurance	14	0.32	0.02 to 0.63	-0.41 to 1.06	0.04	63%	0.48	Very low
Exercise category	Anaerobic performance	18	0.28	0.01 to 0.55	-0.44 to 1.00	0.04	0%	0.48	Very low
Exercise category	Muscular endurance	45	0.15	-0.02 to 0.32	-0.54 to 0.84	0.09	64%	0.48	Low
Exercise category	Strength/power	37	0.10	-0.08 to 0.27	-0.60 to 0.79	0.28	22%	0.48	Low
Timing	40-45 min	21	-0.04	-0.40 to 0.32	-0.77 to 0.70	0.83	0	0.37	Very low
Timing	60 min	90	0.22	0.05 to 0.38	-0.44 to 0.87	0.01	58%	0.37	Low
Timing	120 min	3	-0.04	-0.71 to 0.63	-0.96 to 0.88	0.90	0	0.37	Very low
Dosage	4-4.4 g	2	-0.06	-0.75 to 0.63	-1.06 to 0.94	0.87	0	0.87	Low
Dosage	8 g	86	0.20	0.02 to 0.38	-0.55 to 0.95	0.03	61%	0.87	Low
Dosage	12 g	20	0.08	-0.44 to 0.59	-0.82 to 1.20	0.77	5%	0.87	Low
Dosage	15 g	2	0.08	-0.76 to 0.92	-1.03 to 1.20	0.85	0	0.87	Very low
Form	Pill	1	-0.05	-0.82 to 0.73	-1.05 to 0.96	0.90	0	0.58	Very low
Form	Beverage	113	0.17	0.02 to 0.32	-0.49 to 0.83	0.02	52%	0.58	Low

Table notes: K, the total number of effects included in the pooled effect size; GRADE, Criteria for Certainty of Evidence; Hedges' g, the effect size indicators used in the pooled; 95%CI, 95% confidence interval; PI, prediction Interval; P-value, statistically significant P values for pooled results; p_between for between subgroup difference; I2, quantitative indicators of heterogeneity; Power, exploratory post hoc statistical power estimate for pooled effect size.

Electronic Supplementary Material S7 (ROB2 Assessment Tool for Risk of Bias)

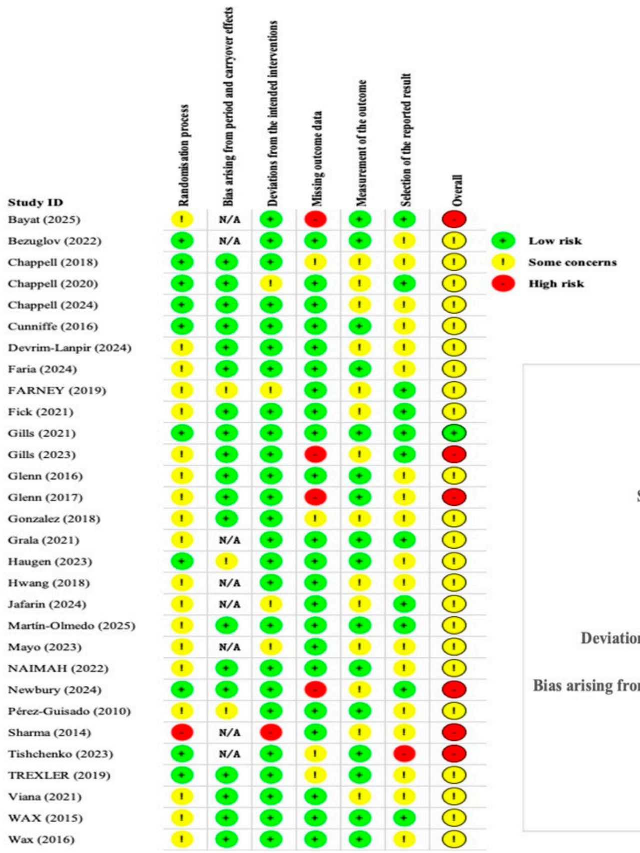

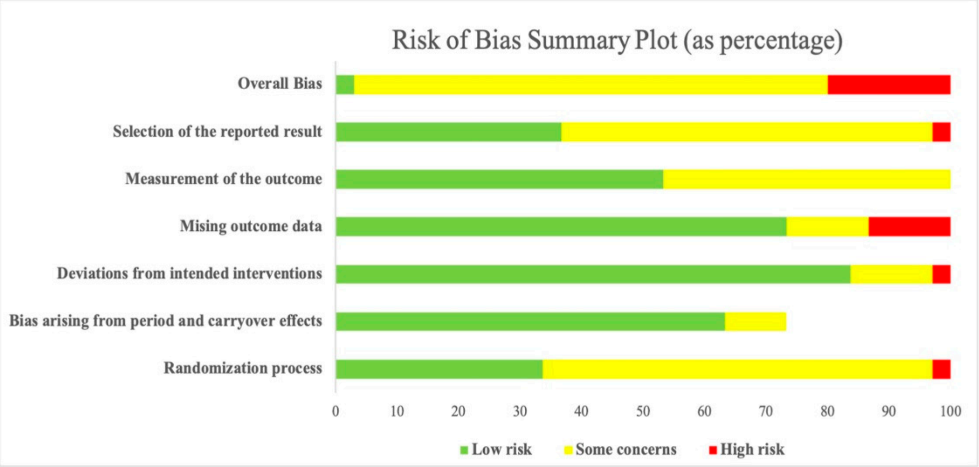

Electronic Supplementary Material S8 (Funnel Plot)

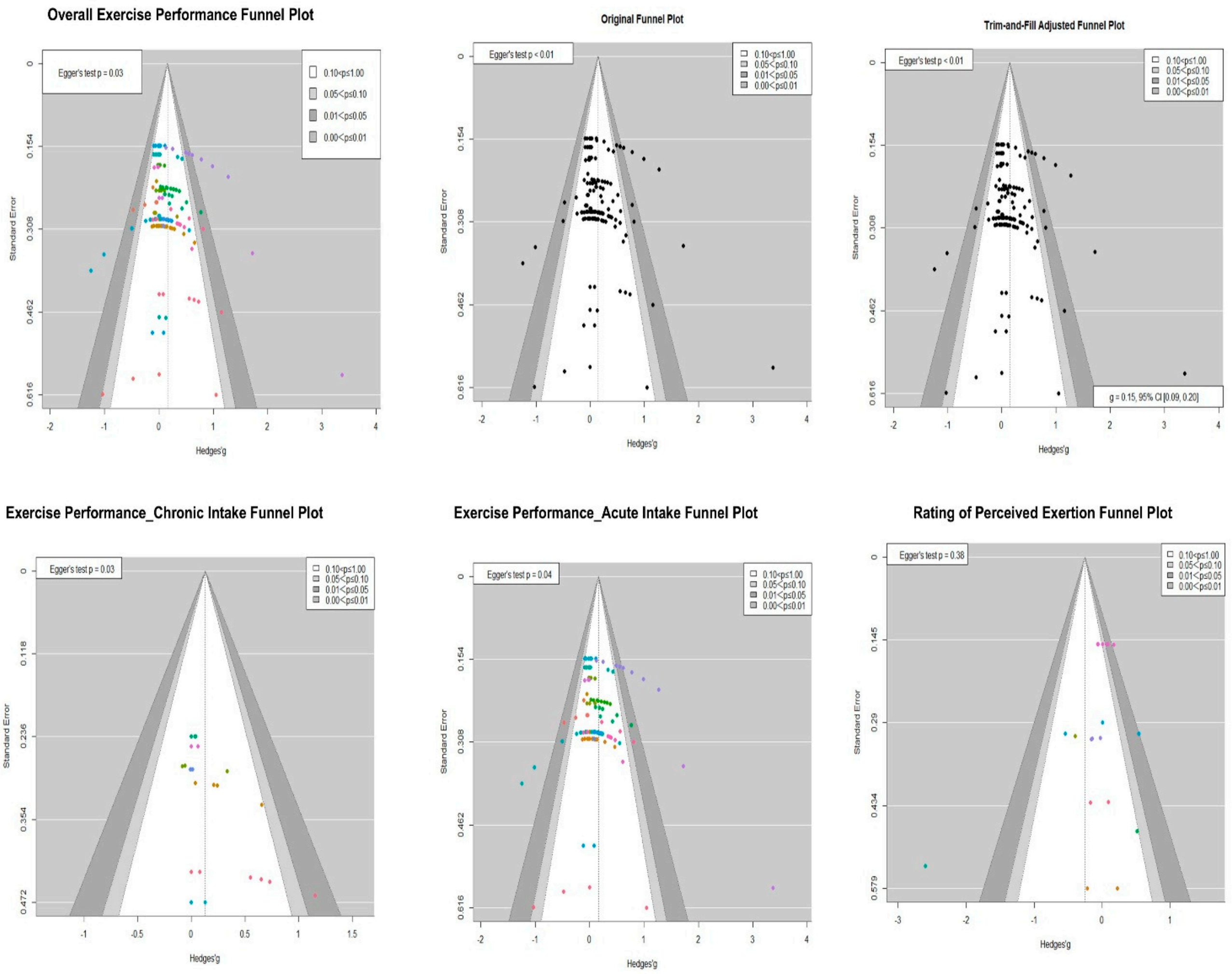

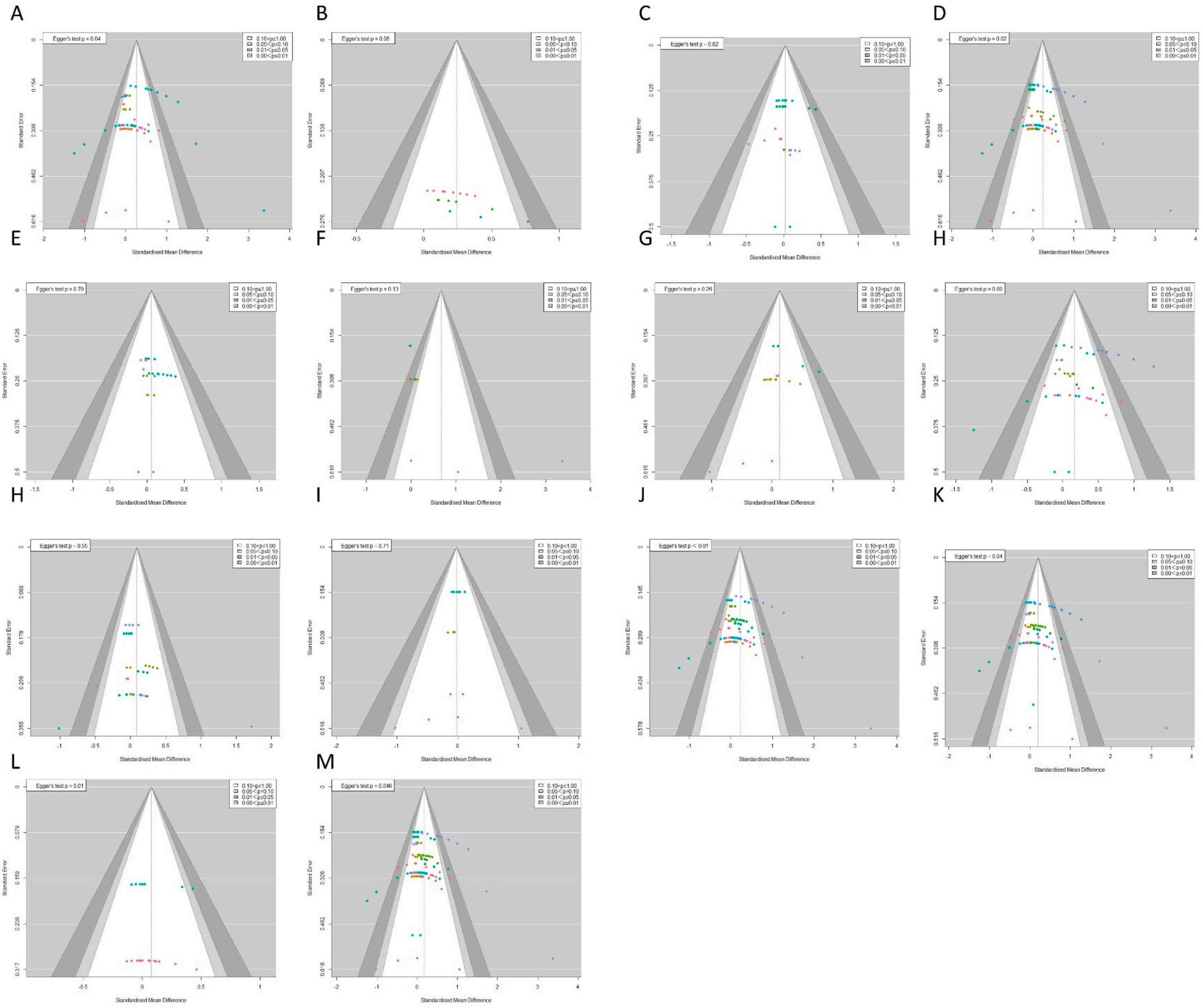

Notes: Colorful circles, represent different effect size points. From A to M, they are male, female, mixed, trained, untrained, aerobic endurance, anaerobic performance, muscular endurance, strength/power, before 40-45 minutes, before 60 minutes, 8 g, 12 g, beverage.

Electronic Supplementary Material S9 (Power Visualization)

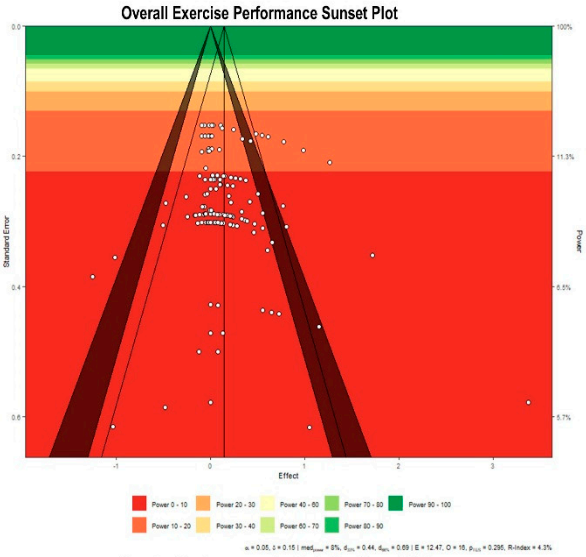

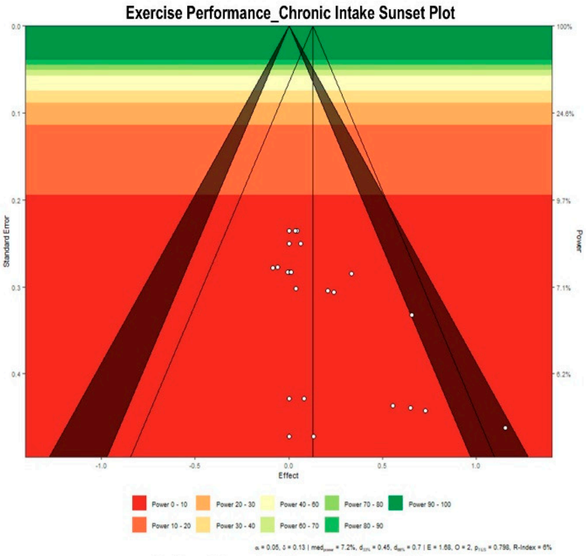

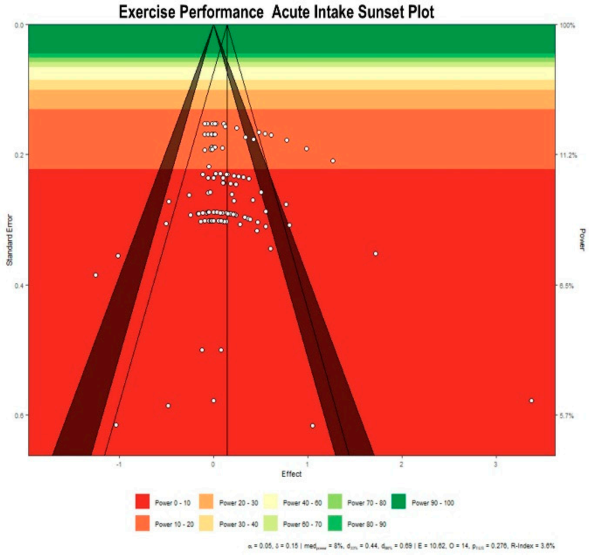

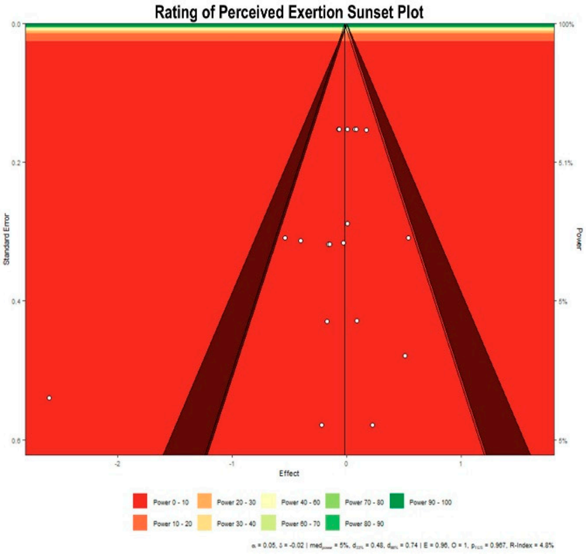

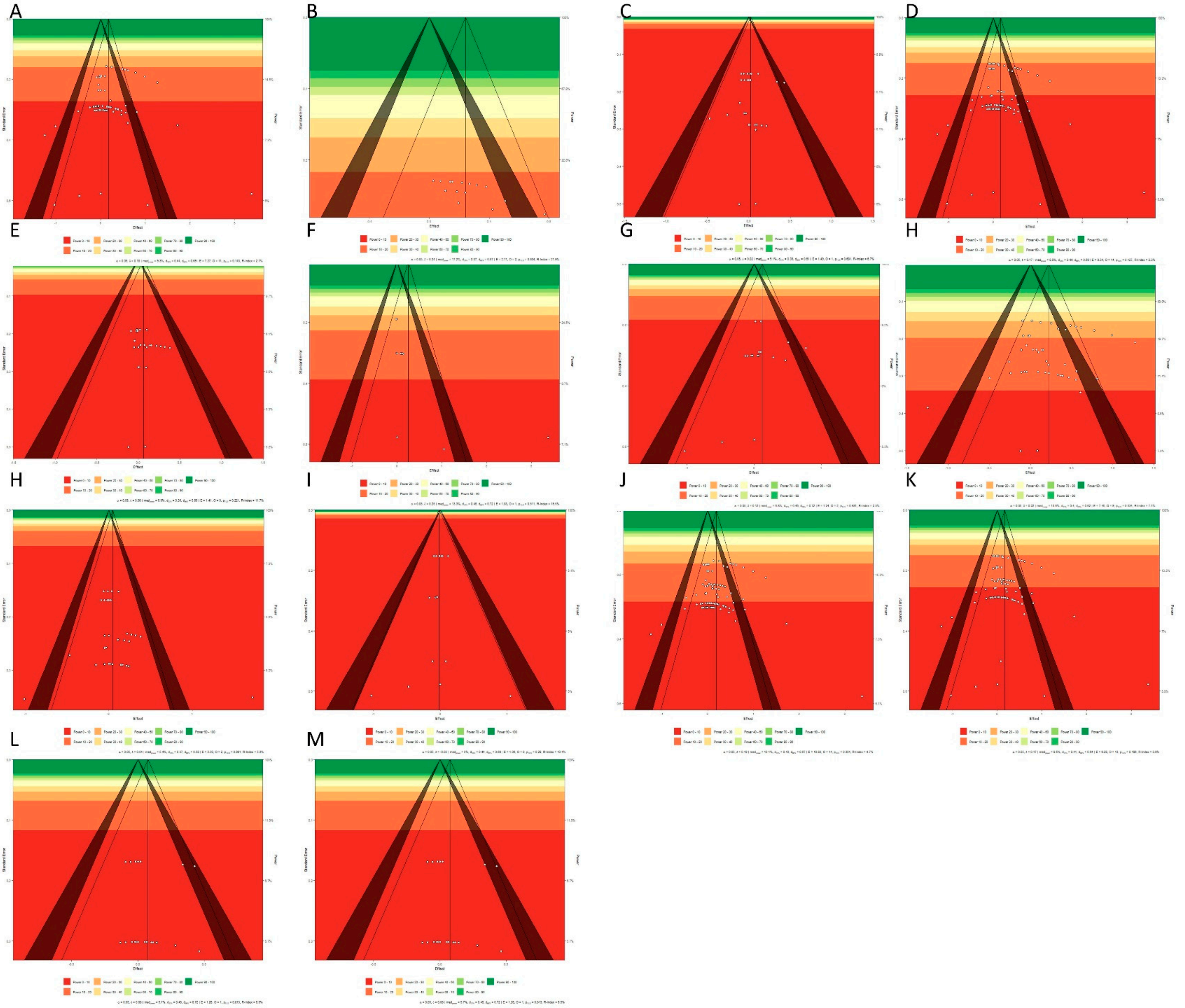

Notes**:** The vertical solid line represents the pooled effect size, and the vertical dash line represents the adjusted pooled effect size. Significance contours at .05 and .01 levels are noted by the shaded area. ***manpower*** indicates the median power of all included effect sizes. ***d33%*** and ***d66%*** indicate the true effect sizes necessary for achieving 33% and 66% levels of median power. ***E, O, and PTES*** show the results of a test of excess significance. ***R-index*** denotes the expected replicability of findings. From A to M, they are male, female, mixed, trained, untrained, aerobic endurance, anaerobic performance, muscular endurance, strength/power, before 40-45 minutes, before 60 minutes, 8 g, 12 g, beverage.

Electronic Supplementary Material S10 (PEDro Assessment)

Study	Item 1	Item 2	Item 3	Item 4	Item 5	Item 6	Item 7	Item 8	Item 9	Item 10	Item 11	Item 12	Total
Bayat et al. (2025)	Yes	Yes	No	Yes	Yes	No	Yes	Yes	No	Yes	Yes	Yes	8
Bezuglov et al. (2022)	Yes	Yes	No	Yes	Yes	Yes	No	Yes	Yes	Yes	Yes	No	8
Chappell et al. (2018)	Yes	Yes	Yes	Yes	Yes	No	No	Yes	Yes	Yes	Yes	No	8
Chappell et al. (2020)	Yes	Yes	Yes	Yes	Yes	Yes	No	Yes	Yes	Yes	Yes	No	9
Chappell et al. (2024)	Yes	Yes	Yes	Yes	Yes	Yes	Yes	Yes	Yes	Yes	Yes	No	10
Cunniffe et al. (2016)	Yes	Yes	No	Yes	Yes	Yes	Yes	Yes	Yes	Yes	Yes	No	9
Devrim-Lanpir et al. (2024)	Yes	Yes	No	Yes	Yes	No	No	Yes	Yes	Yes	Yes	No	7
Faria & Egan (2024)	Yes	Yes	No	Yes	Yes	No	No	Yes	Yes	Yes	Yes	No	7
Farney et al. (2019)	Yes	Yes	No	Yes	Yes	No	No	Yes	Yes	Yes	Yes	No	7
Fick et al. (2021)	Yes	Yes	No	Yes	Yes	No	No	Yes	Yes	Yes	Yes	No	7
Gills et al. (2021)	Yes	Yes	No	Yes	Yes	Yes	Yes	Yes	Yes	Yes	Yes	No	9
Gills et al. (2023)	Yes	No	Yes	Yes	Yes	No	No	No	Yes	Yes	Yes	Yes	7
Glenn et al. (2016)	Yes	No	Yes	No	Yes	Yes	Yes	Yes	No	Yes	Yes	Yes	8
Glenn et al. (2017)	Yes	Yes	Yes	Yes	Yes	No	Yes	No	No	Yes	Yes	No	7
Gonzalez et al. (2018)	Yes	Yes	Yes	No	Yes	No	No	Yes	Yes	Yes	Yes	No	7

Grala et al. (2021)	Yes	Yes	No	Yes	Yes	No	Yes	Yes	No	Yes	Yes	Yes	8
Haugen et al. (2023)	Yes	Yes	Yes	Yes	Yes	No	No	Yes	Yes	Yes	Yes	No	8
Hwang et al. (2018)	Yes	Yes	Yes	Yes	Yes	No	No	Yes	No	Yes	Yes	No	7
Jafari et al. (2024)	Yes	No	No	Yes	No	No	No	Yes	Yes	Yes	Yes	No	5
Martín-Olmedo et al. (2024)	Yes	Yes	No	Yes	Yes	No	No	Yes	Yes	Yes	No	No	6
Mayo et al. (2023)	Yes	Yes	Yes	Yes	No	No	No	Yes	No	Yes	Yes	Yes	7
Naimah et al. (2022)	Yes	Yes	No	Yes	Yes	Yes	Yes	Yes	No	Yes	Yes	No	8
Newbury et al. (2024)	Yes	Yes	Yes	Yes	Yes	No	Yes	No	No	Yes	Yes	Yes	8
Pérez-Guisado & Jakeman (2010)	Yes	Yes	No	Yes	Yes	No	Yes	Yes	Yes	Yes	Yes	No	8
Sharma et al. (2014)	Yes	Yes	No	Yes	No	No	No	Yes	No	Yes	Yes	No	5
Tishchenko et al. (2023)	Yes	Yes	No	Yes	No	Yes	Yes	Yes	No	Yes	Yes	No	7
Trexler et al. (2019)	Yes	Yes	Yes	Yes	Yes	No	No	Yes	Yes	Yes	Yes	No	8
Viana et al. (2021)	Yes	Yes	No	Yes	Yes	No	No	Yes	No	Yes	Yes	No	6
Wax et al. (2015)	Yes	Yes	No	Yes	Yes	No	No	Yes	No	Yes	Yes	Yes	7
Wax et al. (2016)	Yes	Yes	No	Yes	Yes	Yes	Yes	Yes	Yes	Yes	Yes	No	9

Electronic Supplementary Material S11 (Summary of Sensitivity Analysis Results)

Test Methods	K	Hedges' g	95% CI	P	95% PI	I2
**Overall Exercise performance**						
r = 0.8	138	0.19	[0.04, 0.33]	0.01	[-0.64, 1.02]	79%
r = 0.2	138	0.14	[0.05, 0.23]	<0.01	[-0.25, 0.53]	14%
ML	138	0.16	[0.05, 0.26]	<0.01	[-0.35, 0.66]	45%
Remove outliers	130	0.11	[0.04, 0.19]	<0.01	[-0.17, 0.40]	13%
**Overall Moderator: Acute**						
r = 0.8	114	0.19	[0.03, 0.34]	0.02	[-0.66, 1.03]	82%
r = 0.2	114	0.14	[0.04, 0.25]	0.01	[-0.26, 0.54]	22%
ML	114	0.16	[0.04, 0.27]	0.01	[-0.35, 0.67]	52%
Remove outliers	105	0.11	[0.02, 0.20]	0.02	[-0.21, 0.43]	30%
**Overall Moderator: Chronic**						
r = 0.8	24	0.18	[-0.08, 0.45]	0.17	[-0.69, 1.05]	6%
r = 0.2	24	0.14	[-0.06, 0.34]	0.17	[-0.29, 0.57]	0
ML	24	0.15	[-0.06, 0.36]	0.15	[-0.39, 0.69]	0
Remove outliers	17	0.13	[-0.08, 0.35]	0.28	[-0.24, 0.50]	0
**Independent Analysis: Acute**						
r = 0.5	114	0.16	[0.02, 0.30]	0.02	[-0.48, 0.80]	52%
r = 0.8	114	0.19	[0.01, 0.37]	0.04	[-0.75, 1.13]	82%

r = 0.2	114	0.14	[0.03, 0.25]	0.02	[-0.30, 0.58]	22%
ML	114	0.16	[0.03, 0.30]	0.02	[-0.45, 0.77]	52%
Remove outliers	109	0.11	[0.02, 0.21]	0.02	[-0.26, 0.48]	40%
**Independent Analysis: Chronic**						
r = 0.5	24	0.13	[-0.01, 0.28]	0.08	[-0.09, 0.35]	0
r = 0.8	24	0.15	[0.01, 0.30]	0.047	[-0.16, 0.46]	6%
r = 0.2	24	0.13	[-0.03, 0.29]	0.11	[-0.10, 0.36]	0
ML	24	0.13	[-0.01, 0.26]	0.06	[-0.01, 0.27]	0
Remove outliers	22	0.09	[-0.05, 0.22]	0.19	[-0.05, 0.22]	0
**Rating of Perceived Exertion**						
r = 0.8	19	-0.29	[-0.95, -0.37]	0.37	[-0.64, 1.02]	82%
r = 0.2	19	-0.01	[-0.15, 0.12]	0.82	[-0.15, 0.12]	14%
ML	19	-0.24	[-0.70, 0.23]	0.30	[-1.47, 1.00]	49%
Remove outliers	18	0.01	[-0.10, 0.12]	0.87	[-0.10, 0.12]	0

Notes: ***K***, the total number of effects included in the pooled effect size; ***Hedges'g***, the effect size indicators used in the pooled; ***95%CI***, 95% confidence interval; ***95%PI***, Prediction interval, ***P-value***, statistically significant P values for pooled results; I^2, quantitative indicators of heterogeneity; ***Power***, exploratory post hoc statistical power estimate for pooled effect size; ***r***, correlation coefficient between citrulline malate group and placebo group; ***ML***, maximum likelihood estimation.

Electronic Supplementary Material S12 (A Sensitivity Analysis Based on Level-3 Leave-one-out)

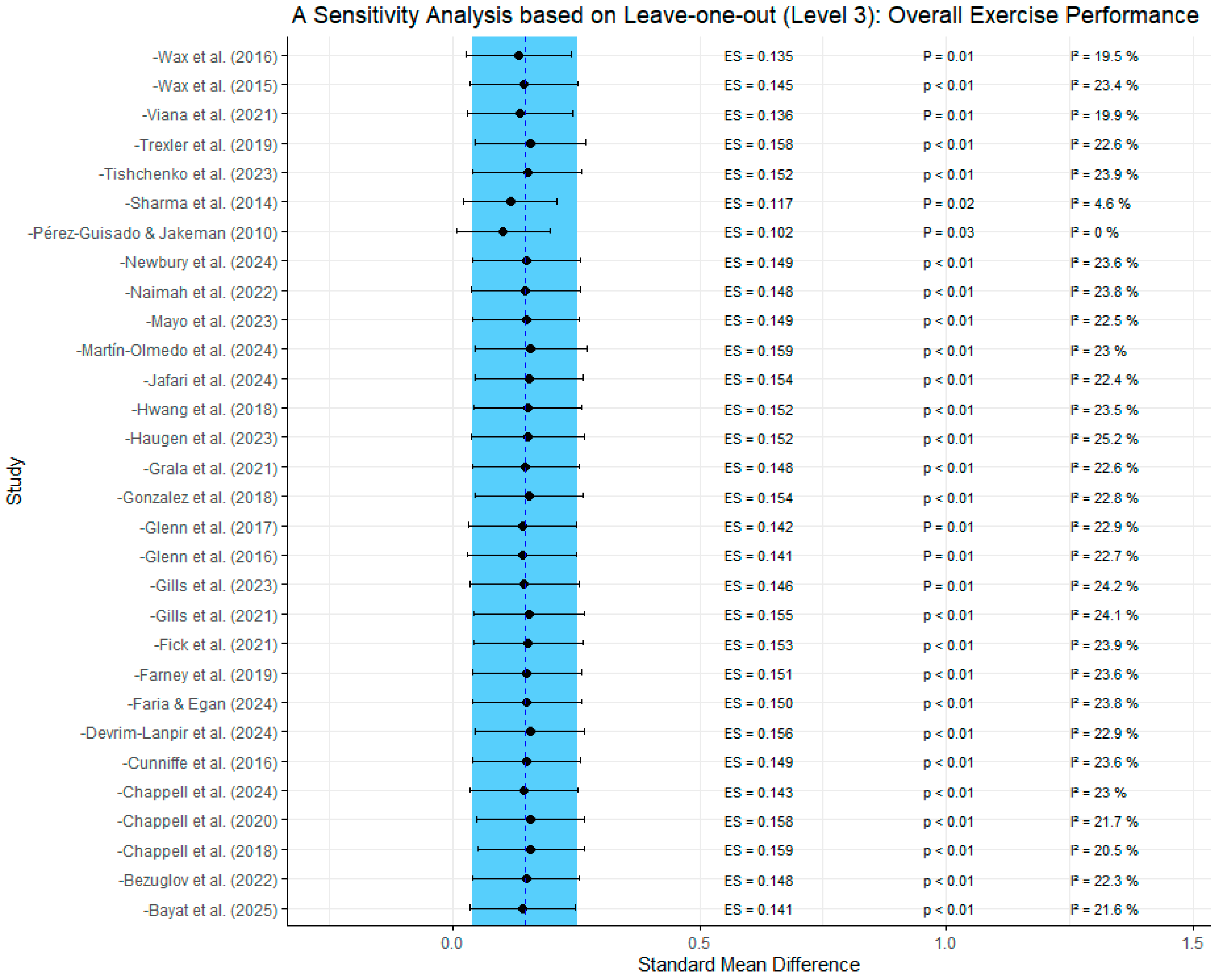

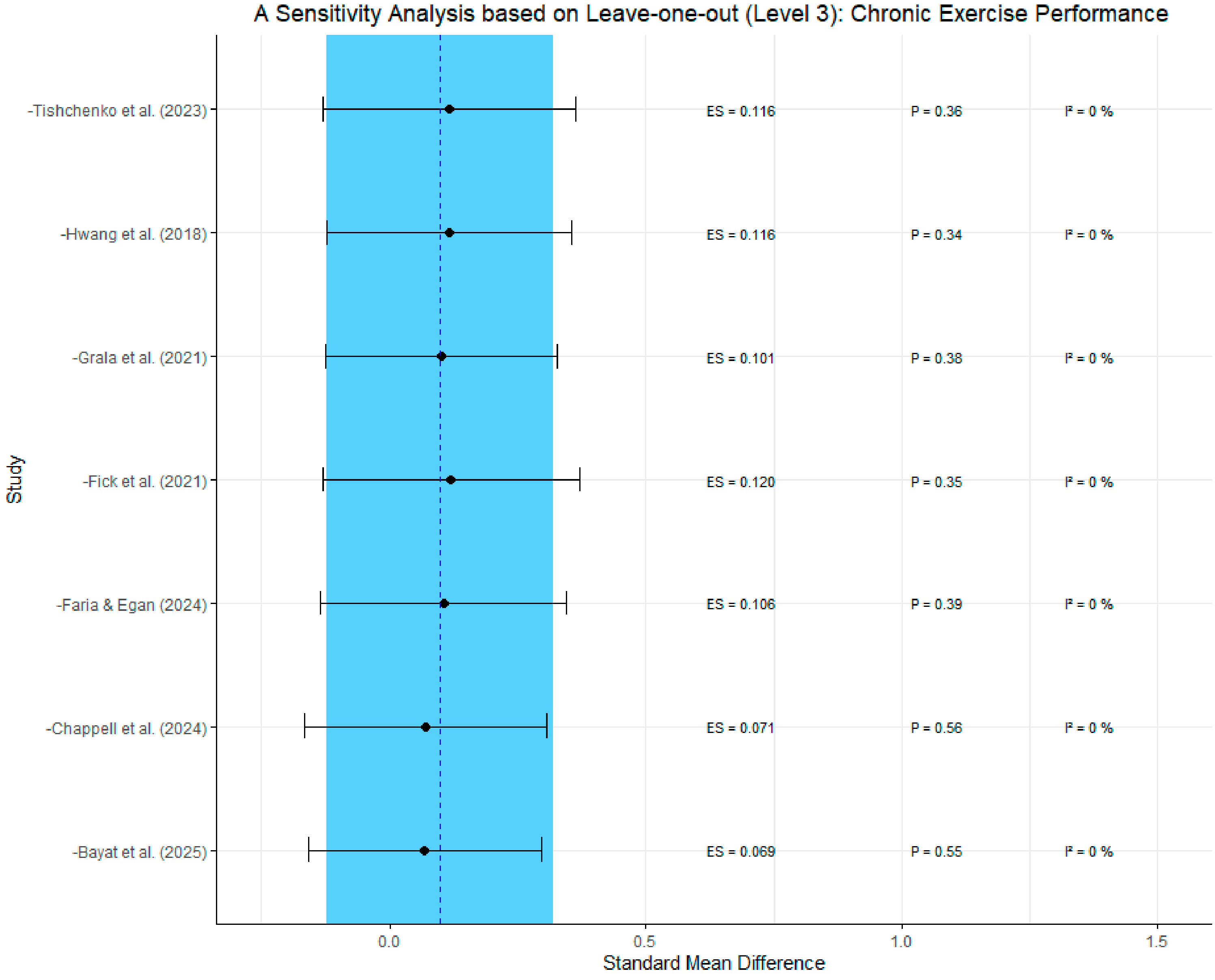
A Sensitivity Analysis based on Leave-one-out (Level 3): Chronic Exercise Performance
Study
-Tishchenko et al. (2023)
-Hwang et al. (2018)
-Grala et al. (2021)
-Fick et al. (2021)
-Faria & Egan (2024)
-Chappell et al. (2024)
-Bayat et al. (2025)
ES = 0.116
ES = 0.116
ES = 0.101
ES = 0.120
ES = 0.106
ES = 0.071
ES = 0.069
P = 0.36
P = 0.34
P = 0.38
P = 0.35
P = 0.39
P = 0.56
P = 0.55
I² = 0 %
I² = 0 %
I² = 0 %
I² = 0 %
I² = 0 %
I² = 0 %
I² = 0 %
0.0
0.5
1.0
1.5
Standard Mean Difference

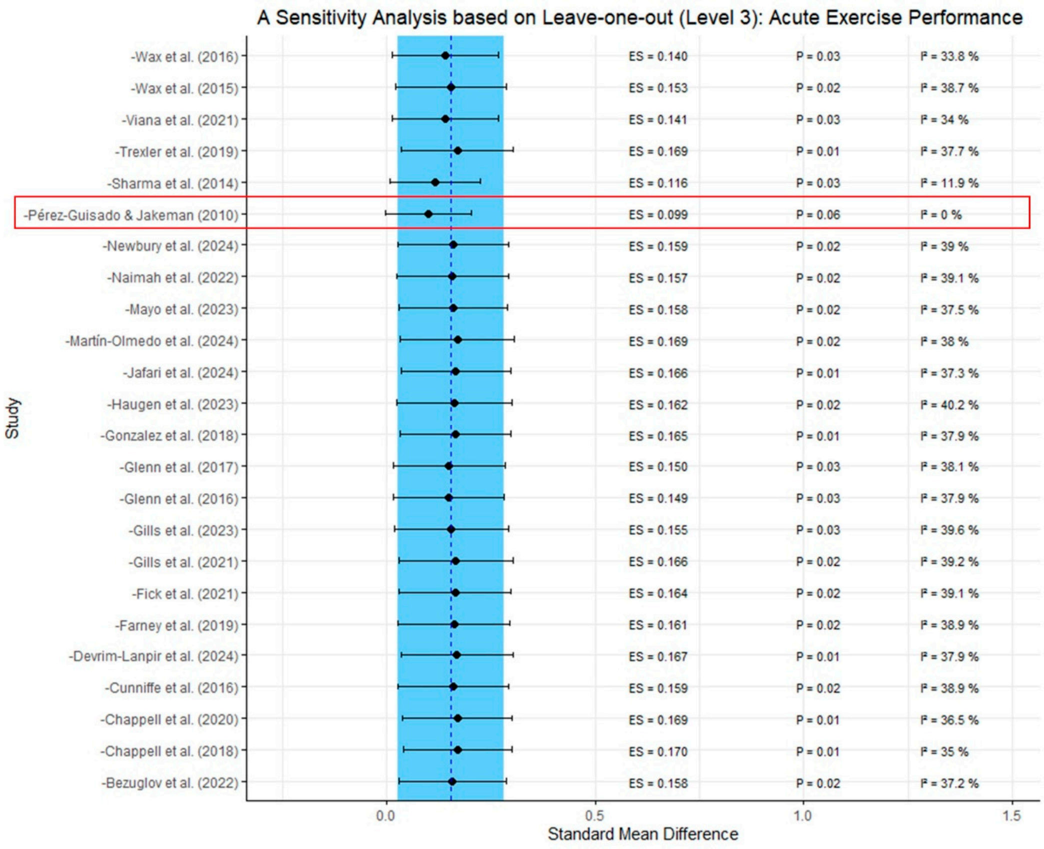
A Sensitivity Analysis based on Leave-one-out (Level 3): Acute Exercise Performance
Study
-Wax et al. (2016) ES = 0.140 P = 0.03 I^2 = 33.8 %
-Wax et al. (2015) ES = 0.153 P = 0.02 I^2 = 38.7 %
-Viana et al. (2021) ES = 0.141 P = 0.03 I^2 = 34 %
-Trexler et al. (2019) ES = 0.169 P = 0.01 I^2 = 37.7 %
-Sharma et al. (2014) ES = 0.116 P = 0.03 I^2 = 11.9 %
-Pérez-Guisado & Jakeman (2010) ES = 0.099 P = 0.06 I^2 = 0 %
-Newbury et al. (2024) ES = 0.159 P = 0.02 I^2 = 39 %
-Naimah et al. (2022) ES = 0.157 P = 0.02 I^2 = 39.1 %
-Mayo et al. (2023) ES = 0.158 P = 0.02 I^2 = 37.5 %
-Martín-Olmedo et al. (2024) ES = 0.169 P = 0.02 I^2 = 38 %
-Jafari et al. (2024) ES = 0.166 P = 0.01 I^2 = 37.3 %
-Haugen et al. (2023) ES = 0.162 P = 0.02 I^2 = 40.2 %
-Gonzalez et al. (2018) ES = 0.165 P = 0.01 I^2 = 37.9 %
-Glenn et al. (2017) ES = 0.150 P = 0.03 I^2 = 38.1 %
-Glenn et al. (2016) ES = 0.149 P = 0.03 I^2 = 37.9 %
-Gills et al. (2023) ES = 0.155 P = 0.03 I^2 = 39.6 %
-Gills et al. (2021) ES = 0.166 P = 0.02 I^2 = 39.2 %
-Fick et al. (2021) ES = 0.164 P = 0.02 I^2 = 39.1 %
-Farney et al. (2019) ES = 0.161 P = 0.02 I^2 = 38.9 %
-Devrim-Lanpir et al. (2024) ES = 0.167 P = 0.01 I^2 = 37.9 %
-Cunniffe et al. (2016) ES = 0.159 P = 0.02 I^2 = 38.9 %
-Chappell et al. (2020) ES = 0.169 P = 0.01 I^2 = 36.5 %
-Chappell et al. (2018) ES = 0.170 P = 0.01 I^2 = 35 %
-Bezuglov et al. (2022) ES = 0.158 P = 0.02 I^2 = 37.2 %
0.0
0.5
1.0
1.5
Standard Mean Difference

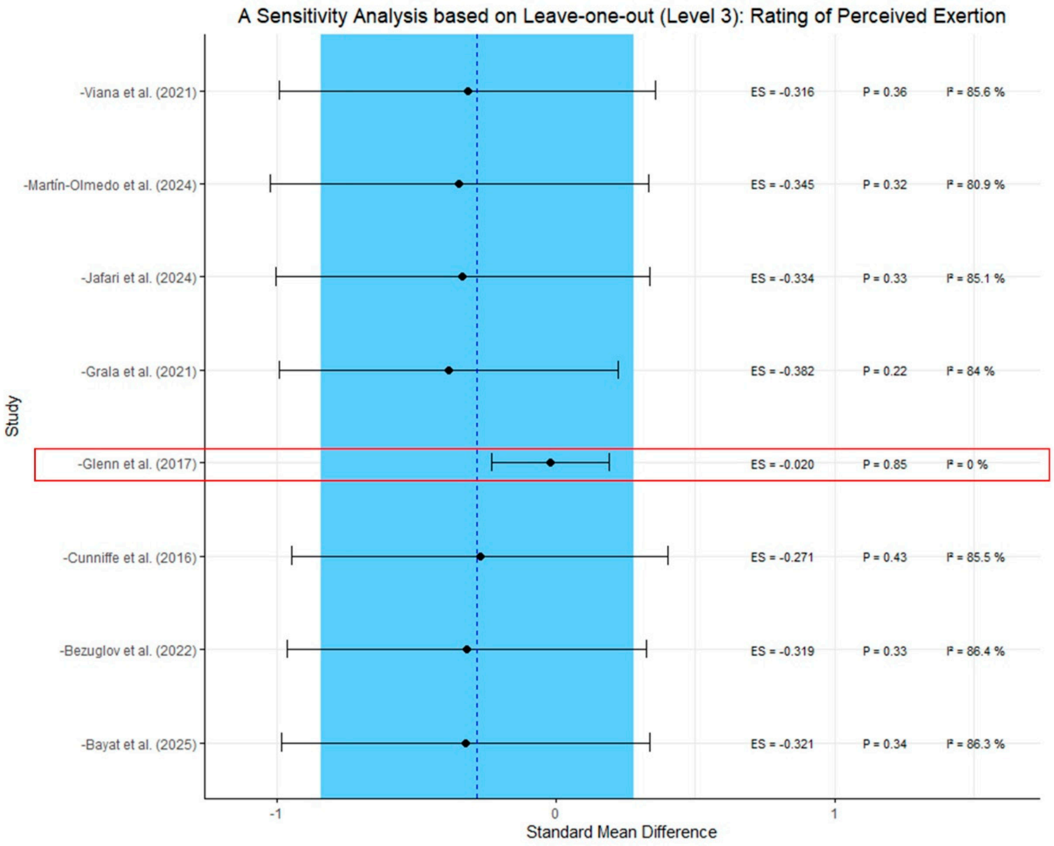
A Sensitivity Analysis based on Leave-one-out (Level 3): Rating of Perceived Exertion
Study
-Viana et al. (2021) ES = -0.316 P = 0.36 I^2 = 85.6 %
-Martín-Olmedo et al. (2024) ES = -0.345 P = 0.32 I^2 = 80.9 %
-Jafari et al. (2024) ES = -0.334 P = 0.33 I^2 = 85.1 %
-Grala et al. (2021) ES = -0.382 P = 0.22 I^2 = 84 %
-Glenn et al. (2017) ES = -0.020 P = 0.85 I^2 = 0 %
-Cunniffe et al. (2016) ES = -0.271 P = 0.43 I^2 = 85.5 %
-Bezuglov et al. (2022) ES = -0.319 P = 0.33 I^2 = 86.4 %
-Bayat et al. (2025) ES = -0.321 P = 0.34 I^2 = 86.3 %
-1
0
1
Standard Mean Difference

Electronic Supplementary Material S13 (Moderator Analysis After Excluding Outliers)

Subgroup	k	g [95% CI]		P_{within}	Forest plot	I^2	$P_{between}$
**Sex**						**0.37**	**0.35**
Male	65	0.12 [0.01, 0.26]	*	0.046			
Female	14	0.24 [-0.03, 0.52]		0.08			
Mixed	28	0.02 [-0.15, 0.19]		0.84			
**Trained Status**						**0.41**	**0.58**
Trained	86	0.14 [0.02, 0.25]	*	0.02			
Untrained	20	0.07 [-0.14, 0.28]		0.51			
**Exercise Type**						**0.34**	**0.89**
Aerobic Endurance	11	0.09 [-0.20, 0.38]		0.56			
Anaerobic Performance	14	0.01 [-0.26, 0.28]		0.96			
Muscular Endurance	43	0.12 [-0.01, 0.24]		0.06			
Strength/Power	36	0.10 [-0.03, 0.22]		0.13			
**Supplementation Timing**						**0.36**	**0.14**
Before 40-45 minutes	19	-0.04 [-0.28, 0.20]		0.74			
Before 60 minutes	85	0.15 [0.05, 0.26]	*	0.01			
**CM dosage**						**0.44**	**0.78**
8 g	82	0.12 [0.01, 0.23]	*	0.04			
12 g	20	0.08 [-0.21, 0.36]		0.60			
**CM Form**						**0.40**	**N/A**
Beverage	109	0.11 [0.02, 0.21]	*	0.02			

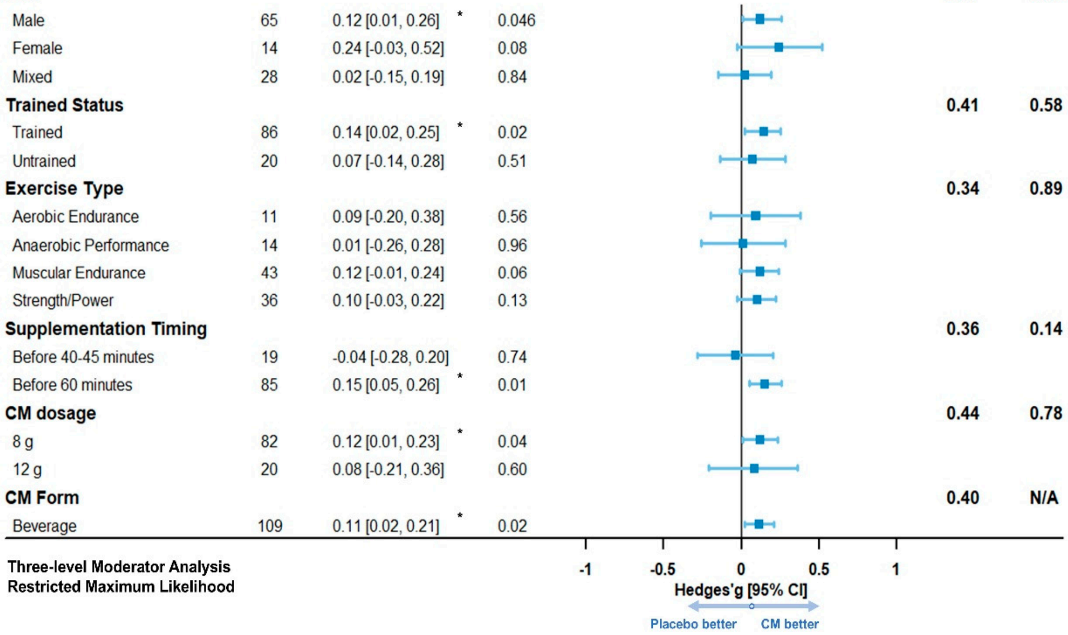

Three-level Moderator Analysis
Restricted Maximum Likelihood

Notes: CM, Citrulline malate; ***K,*** the total number of effects included in the pooled effect size; ***Hedges' g***, the effect size indicators used in the pooled; ***95%CI***, 95% confidence interval; $P_{between}$, statistically significant P values for pooled effect between moderator; P_{within}, statistically significant P values for specific pooled effect of moderator; I^2, quantitative indicators of heterogeneity; ⋆., Represents significant, $p < 0.05$.

Electronic Supplementary Material S14 (Regression Analysis After Excluding Outliers)

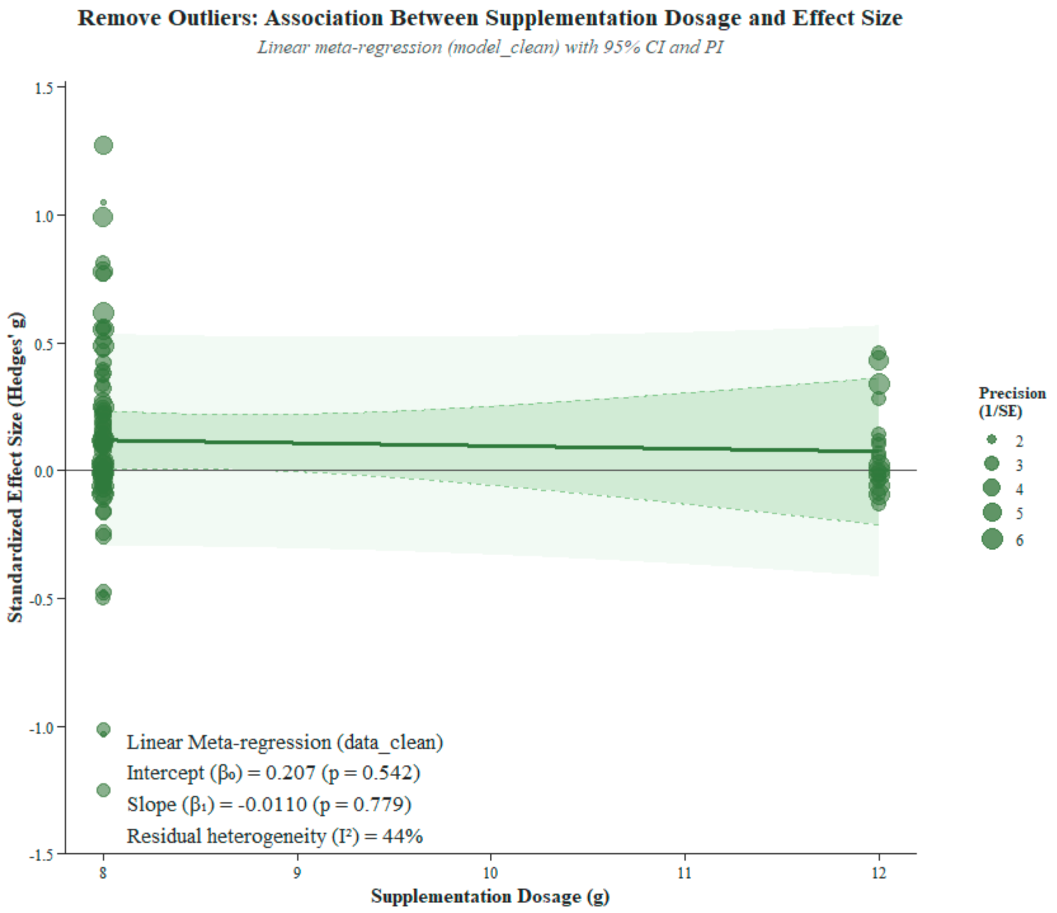

Notes: β0 represents the intercept; ***β1***, ***β2***, and ***β3*** represent the slopes; ***Hedges' g***, the effect size indicators used in the pooled; ***I^2*** means heterogeneity.

Reference

Bayat, D., Azizi, M., Behpour, N., & Tinsley, G. M. (2025). Changes in resistance training performance, rating of perceived exertion, and blood biomarkers after six weeks of supplementation with L-citrulline vs. L-citrulline DL-malate in resistance-trained men: A double-blind placebo-controlled trial. *Journal of the International Society of Sports Nutrition*, *22*(1), 2513944. https://doi.org/10.1080/15502783.2025.2513944

Bezuglov, E., Morgans, R., Lazarev, A., Kalinin, E., Butovsky, M., Savin, E., Tzgoev, E., Pirmakhanov, B., Emanov, A., Zholinsky, A., & Talibov, O. (2022). The effect of a single dose of citrulline on the physical performance of soccer-specific exercise in adult elite soccer players (A pilot randomized double-Blind trial). *Nutrients*, *14*(23), 5036. https://doi.org/10.3390/nu14235036

Chappell, A. J., Allwood, D. M., Johns, R., Brown, S., Sultana, K., Anand, A., & Simper, T. (2018). Citrulline malate supplementation does not improve German volume training performance or reduce muscle soreness in moderately trained males and females. *Journal of the International Society of Sports Nutrition*, *15*(1), 42. https://doi.org/10.1186/s12970-018-0245-8

Chappell, A. J., Allwood, D. M., & Simper, T. N. (2020). Citrulline malate fails to improve German volume training performance in healthy young men and women. *Journal of Dietary Supplements*, *17*(3), 249–260. https://doi.org/10.1080/19390211.2018.1513433

Chappell, A. J., Parry, A., & Simper, T. (2024). The acute effect of citrulline malate loading in resistance trained males on: Anaerobic muscular endurance, force recovery and

muscle soreness. *Journal of Science in Sport and Exercise*. https://doi.org/10.1007/s42978-024-00288-7

Cunniffe, B., Papageorgiou, M., O'Brien, B., Davies, N. A., Grimble, G. K., & Cardinale, M. (2016). Acute citrulline-malate supplementation and high-intensity cycling performance. *The Journal of Strength & Conditioning Research*, *30*(9), 2638. https://doi.org/10.1519/JSC.0000000000001338

Devrim-Lanpir, A., Ihász, F., Demcsik, M., Horváth, A. C., Góczán, P., Czepek, P., Takács, J., Kimble, R., Zare, R., Gunes, F. E., Knechtle, B., Weiss, K., Rosemann, T., & Heinrich, K. M. (2024). Effects of acute citrulline malate supplementation on CrossFit® exercise performance: A randomized, double-blind, placebo-controlled, cross-over study. *Nutrients*, *16*(19), Article 19. https://doi.org/10.3390/nu16193235

Faria, V. S., & Egan, B. (2024). Effects of 3 days of citrulline malate supplementation on short-duration repeated sprint running performance in male team sport athletes. *European Journal of Sport Science*, *24*(6), 758–765. https://doi.org/10.1002/ejsc.12090

Farney, T. M., Bliss, M. V., Hearon, C. M., & Salazar, D. A. (2019). The effect of citrulline malate supplementation on muscle fatigue among healthy participants. *The Journal of Strength & Conditioning Research*, *33*(9), 2464. https://doi.org/10.1519/JSC.0000000000002356

Fick, A. N., Kowalsky, R. J., Stone, M. S., Hearon, C. M., & Farney, T. M. (2021). *Acute and chronic citrulline malate supplementation on muscle contractile properties and fatigue rate of the quadriceps*. https://doi.org/10.1123/ijsnem.2021-0117

Gills, J. L., Glenn, J. M., Gray, M., Romer, B., & Lu, H. (2021). Acute citrulline-malate supplementation is ineffective during aerobic cycling and subsequent anaerobic performance in recreationally active males. *European Journal of Sport Science*, *21*(1), 77–83. https://doi.org/10.1080/17461391.2020.1722757

Gills, J. L., Spliker, B., Glenn, J. M., Szymanski, D., Romer, B., Lu, H.-C., & Gray, M. (2023). Acute citrulline-malate supplementation increases total work in short lower-body isokinetic tasks for recreationally active females during menstruation. *The Journal of Strength & Conditioning Research*, *37*(6), 1225. https://doi.org/10.1519/JSC.0000000000004095

Glenn, J. M., Gray, M., Jensen, A., Stone, M. S., & Vincenzo, J. L. (2016). Acute citrulline-malate supplementation improves maximal strength and anaerobic power in female, masters athletes tennis players. *European Journal of Sport Science*, *16*(8), 1095–1103. https://doi.org/10.1080/17461391.2016.1158321

Glenn, J. M., Gray, M., Wethington, L. N., Stone, M. S., Stewart, R. W., & Moyen, N. E. (2017). Acute citrulline malate supplementation improves upper- and lower-body submaximal weightlifting exercise performance in resistance-trained females. *European Journal of Nutrition*, *56*(2), 775–784. https://doi.org/10.1007/s00394-015-1124-6

Gonzalez, A. M., Spitz, R. W., Ghigiarelli, J. J., Sell, K. M., & Mangine, G. T. (2018). Acute effect of citrulline malate supplementation on upper-body resistance exercise performance in recreationally resistance-trained men. *The Journal of Strength & Conditioning Research*, *32*(11), 3088.

https://doi.org/10.1519/JSC.0000000000002373

Grala, A., Candellório, É., Sperandio, P., Maldonado, E., Anjos, B. dos, Jacinto, J., Casonatto, J., & Aguiar, A. (2021). Effects of citrulline malate supplementation on aerobic and muscular endurance in young adults men. *Journal of Health Sciences*, *23*(1), Article 1. https://doi.org/10.17921/2447-8938.2021v23n1p72-78

Haugen, M. E., Vårvik, F. T., Grgic, J., Studsrud, H., Austheim, E., Zimmermann, E. M., Falch, H. N., Larsen, S., van den Tillaar, R., & Bjørnsen, T. (2023). Effect of isolated and combined ingestion of caffeine and citrulline malate on resistance exercise and jumping performance: A randomized double-blind placebo-controlled crossover study. *European Journal of Nutrition*, *62*(7), 2963–2975. https://doi.org/10.1007/s00394-023-03212-x

Hwang, P., Morales Marroquín, F. E., Gann, J., Andre, T., McKinley-Barnard, S., Kim, C., Morita, M., & Willoughby, D. S. (2018). Eight weeks of resistance training in conjunction with glutathione and L-Citrulline supplementation increases lean mass and has no adverse effects on blood clinical safety markers in resistance-trained males. *Journal of the International Society of Sports Nutrition*, *15*(1), 30. https://doi.org/10.1186/s12970-018-0235-x

Jafari, R. A., Hosseini, S. R. A., Rashidlamir, A., & Nobari, H. (2024). Evaluating the impact of active and passive recovery strategies and citrulline-malate supplementation in wrestling: Do the results add up? *Acta Kinesiologica*, *18*(2), 58–69.

Martín-Olmedo, J. J., Miras-Moreno, S., Cuadra-Montes, K., García-Ramos, A., Ruiz, J. R., & Jurado-Fasoli, L. (2024). *Malate or not? Acute effects of L-citrulline versus*

citrulline malate on neuromuscular performance in young, trained adults: A randomized, double-blind, placebo-controlled crossover trial. https://doi.org/10.1123/ijsnem.2024-0006

Mayo, J., Lyons, B. C., Tucker, W. S., & Wax, B. (2023). Acute citrulline malate supplementation does not improve anaerobic capacity in healthy young adults: A pilot study. *Journal of Exercise and Nutrition*, *6*(1). https://doi.org/10.53520/jen2023.103149

Naimah, N. M., Linoby, A., Norhamazi, I., Haslan, A. N., Zubir, S. M. S., Noor, M. A. M., & Zamri, F. N. S. (2022). Influence of acute ctrulline-malate supplementation on maximal strength and anaerobic power in combat athletes. *Journal of Physical Education and Sport*, *22*(9), 2083–2088. https://doi.org/10.7752/jpes.2022.09266

Newbury, J. W., Cole, M., Bailey, S. J., Kelly, A. L., & Gough, L. A. (2024). Citrulline malate fails to improve repeated 300 m swimming times in highly trained swimmers. *Physiologia*, *4*(2), Article 2. https://doi.org/10.3390/physiologia4020014

Pérez-Guisado, J., & Jakeman, P. M. (2010). Citrulline malate enhances athletic anaerobic performance and relieves muscle soreness. *The Journal of Strength & Conditioning Research*, *24*(5), 1215. https://doi.org/10.1519/JSC.0b013e3181cb28e0

Sharma, N., Shori, G., & Jaipuriar, D. S. (2014). *Effects of single dose of citrulline malate on performance in collegiate male athltes*. *20*(33).

Tishchenko, A. A., Kaplanyan, D. A., Krechetova, V. A., Frantsuzov, Y. K., Shakhbanov, I. S., & Chnavayan, A. V. (2023). Effectiveness of the use of citrulline malate to increase the powerlifters adaptive potential and physical performance. *Journal of Biochemical*

Technology, *14*(2–2023), Article 2–2023. https://doi.org/10.51847/Gd16A1JnMz

Trexler, E. T., Keith, D. S., Schwartz, T. A., Ryan, E. D., Stoner, L., Persky, A. M., & Smith-Ryan, A. E. (2019). Effects of citrulline malate and beetroot juice supplementation on blood flow, energy metabolism, and performance during maximum effort leg extension exercise. *The Journal of Strength & Conditioning Research*, *33*(9), 2321. https://doi.org/10.1519/JSC.0000000000003286

Viana, J. C. B., Azevedo, A. P., Freire de Almeida, R., Vicentini, G., Barauna, V. G., & Guimaraes-Ferreira, L. (2021). Acute citrulline-malate ingestion does not enhance performance in judo athletes. *Idō Movement for Culture*, *21*(1). https://www.proquest.com/docview/2625227318/abstract/987103710F3F444FPQ/1

Wax, B., Kavazis, A. N., & Luckett, W. (2016). Effects of supplemental citrulline-malate Ingestion on blood lactate, cardiovascular dynamics, and resistance exercise performance in trained males. *Journal of Dietary Supplements*, *13*(3), 269–282. https://doi.org/10.3109/19390211.2015.1008615

Wax, B., Kavazis, A. N., Weldon, K., & Sperlak, J. (2015). Effects of supplemental citrulline malate ingestion during repeated bouts of lower-body exercise in advanced weightlifters. *The Journal of Strength & Conditioning Research*, *29*(3), 786. https://doi.org/10.1519/JSC.0000000000000670
